# Modulation of the extracellular matrix by *Streptococcus gallolyticus* subsp. *gallolyticus* and importance in cell proliferation

**DOI:** 10.1371/journal.ppat.1010894

**Published:** 2022-10-03

**Authors:** Ritesh Kumar, John Culver Taylor, Antrix Jain, Sung Yun Jung, Victor Garza, Yi Xu

**Affiliations:** 1 Center for Infectious and Inflammatory Diseases, Institute of Biosciences and Technology, Texas A&M Health Science Center, Houston, Texas, United States of America; 2 MS Proteomics Core, Baylor College of Medicine, Houston, Texas, United States of America; 3 Department of Biochemistry and Molecular Biology, Baylor College of Medicine, Houston, Texas, United States of America; 4 Department of Microbial Pathogenesis and Immunology, College of Medicine, Texas A&M Health Science Center, College Station, Texas, United States of America; 5 Department of Microbiology and Molecular Genetics, McGovern Medical School, UT Health, Houston, Texas, United States of America; Lunds universitet Medicinska fakulteten, SWEDEN

## Abstract

*Streptococcus gallolyticus* subspecies *gallolyticus* (*Sgg*) has a strong clinical association with colorectal cancer (CRC) and actively promotes the development of colon tumors. Previous work showed that this organism stimulates CRC cells proliferation and tumor growth. However, the molecular mechanisms underlying these activities are not well understood. Here, we found that *Sgg* upregulates the expression of several type of collagens in HT29 and HCT116 cells, with type VI collagen (ColVI) being the highest upregulated type. Knockdown of ColVI abolished the ability of *Sgg* to induce cell proliferation and reduced the adherence of *Sgg* to CRC cells. The extracellular matrix (ECM) is an important regulator of cell proliferation. Therefore, we further examined the role of decellularized matrix (dc-matrix), which is free of live bacteria or cells, in *Sgg*-induced cell proliferation. Dc-matrix prepared from *Sgg*-treated cells showed a significantly higher pro-proliferative activity than that from untreated cells or cells treated with control bacteria. On the other hand, dc-matrix from *Sgg*-treated ColVI knockdown cells showed no difference in the capacity to support cell proliferation compared to that from untreated ColVI knockdown cells, suggesting that the ECM by itself is a mediator of *Sgg*-induced cell proliferation. Furthermore, *Sgg* treatment of CRC cells but not ColVI knockdown CRC cells resulted in significantly larger tumors *in vivo*, suggesting that ColVI is important for *Sgg* to promote tumor growth *in vivo*. These results highlight a dynamic bidirectional interplay between *Sgg* and the ECM, where *Sgg* upregulates collagen expression. The *Sgg*-modified ECM in turn affects the ability of *Sgg* to adhere to host cells and more importantly, acts as a mediator for *Sgg*-induced CRC cell proliferation. Taken together, our results reveal a novel mechanism in which *Sgg* stimulates CRC proliferation through modulation of the ECM.

## Author summary

Colorectal cancer (CRC) is a leading cause of cancer-related death. The development of CRC can be strongly influenced by specific gut microbes. Understanding how gut microbes modulate CRC is critical to developing novel strategies to improve clinical diagnosis and treatment of this disease. *S*. *gallolyticus* subsp. *gallolyticus* (*Sgg*) has a strong clinical association with CRC and actively promotes the development of colon tumors. However, the mechanisms *Sgg* utilizes to promote tumors are not well understood. Our results showed for the first time a dynamic interplay between *Sgg* and the extracellular matrix. We found that *Sgg* upregulates the expression of collagens which in turn affects the interaction between *Sgg* and CRC cells and mediates CRC cell proliferation. These findings draw attention to a previously unrecognized dynamic bidirectional interplay between a CRC-associated microbe and the extracellular matrix (ECM). Given the importance of the ECM in normal homeostasis and in tumor microenvironment, these findings have important implications in the context of microbial contribution to cancer.

## Introduction

*Streptococcus gallolyticus* subsp. *gallolyticus* (*Sgg*) belongs to the *S*. *bovis* group of organisms and was previously known as *S*. *bovis* biotype I [1]. It is an opportunistic pathogen that causes bacteremia and infective endocarditis (IE) [2]. *Sgg* is also known to associate with CRC as documented by numerous case reports and case series over the past several decades [3–7]. A meta-analysis study of case reports and case series published up to 2011 found that among *S*. *bovis*-infected patients who underwent colonic evaluation, ~60% had concomitant colon adenomas/carcinomas [8]. Furthermore, patients with *Sgg* bacteremia/IE have a higher risk (~ 7 fold) for CRC compared to bacteremia/IE caused by other species in the *S*. *bovis* group [8], suggesting the existence of a *Sgg*-specific mechanism that promotes the strong association between *Sgg* and CRC. The prevalence of *Sgg* in CRC patients has not been investigated as extensively as the risk for CRC among patients with active *Sgg* infections. Several recent studies showed that *Sgg* was enriched in tumor tissues from CRC patients, suggesting its potential as a biomarker for CRC, while some other studies found no significant association between *Sgg* and CRC [2,9–11].

In addition to the strong clinical association between *Sgg* and CRC, studies have shown that certain *Sgg* strains stimulate the proliferation of CRC cells (designated PP-*Sgg* for proliferation-promoting *Sgg*) and promote the development of tumors in experimental models of CRC [10,12–15]. PP-*Sgg* treatment of human CRC cells led to larger tumors compared to untreated cells in a xenograft model. In an azoxymethane (AOM)-induced CRC model, mice orally gavaged with *Sgg* TX20005, a prototypic PP-*Sgg* strain, had significantly higher tumor burden and dysplasia grade compared to control mice. In a colitis-associated CRC model, oral gavage of *Sgg* augmented tumorigenesis in the colon. Taken together, long-standing clinical observations and recent functional studies indicate that *Sgg* not only has a strong association with CRC but also actively promotes the development of CRC. The mechanism underlying the tumor-promoting activity of *Sgg*, however, is poorly understood. The ability of *Sgg* to stimulate CRC cell proliferation is an important aspect of the tumor-promoting effect of *Sgg*. The Wnt/β-catenin signaling pathway regulates cell fate and proliferation and is a critical pathway in colon tumorigenesis. Previous results indicated that TX20005 induced upregulation of β-catenin and increased nuclear translocation of β-catenin, and that β-catenin signaling was required for *Sgg* to stimulate CRC cell proliferation and tumor growth [10]. The signaling events that lead to *Sgg*-induced activation of β-catenin signaling and cell proliferation were unknown.

The extracellular matrix (ECM) regulates fundamental cell behavior such as cell proliferation, adhesion and migration and plays important roles during normal development as well as in pathological conditions such as cancer [16,17]. The ECM is an important constituent of the tumor microenvironment. Altered ECM composition, structure and mechanical property are common features in tumor tissues and contribute to tumor progression [18–23]. In CRC, multiple studies have found that various types of collagens are upregulated in tumors compared to matched normal tissues [24–31]. Whether gut microbes can provide exogenous signals to modulate ECM expression and dynamics was unknown.

In this study, we found that *Sgg* TX20005 upregulates the expression of collagen *in vitro* and *in vivo*. We demonstrated that upregulation of collagen by TX20005 is important for *Sgg*-induced CRC cell proliferation, upregulation of β-catenin, and tumor growth. Moreover, we demonstrated a direct effect of the ECM in *Sgg*-mediated CRC cell proliferation by using decellularized matrix (dc-matrix) from CRC cells cultured under various treatment conditions. Altogether, our results suggest a novel mechanism in which *Sgg* actively regulates the expression of ECM molecules which in turn affects the ability of *Sgg* to stimulate CRC cell proliferation in a direct and indirect manner. This mechanism has important implications in the context of microbial contribution to CRC and may be important to *Sgg* IE.

## Results

### *Sgg* increases collagen expression in human CRC cells and in colonic tissues *in vivo*

**Fig 1:**
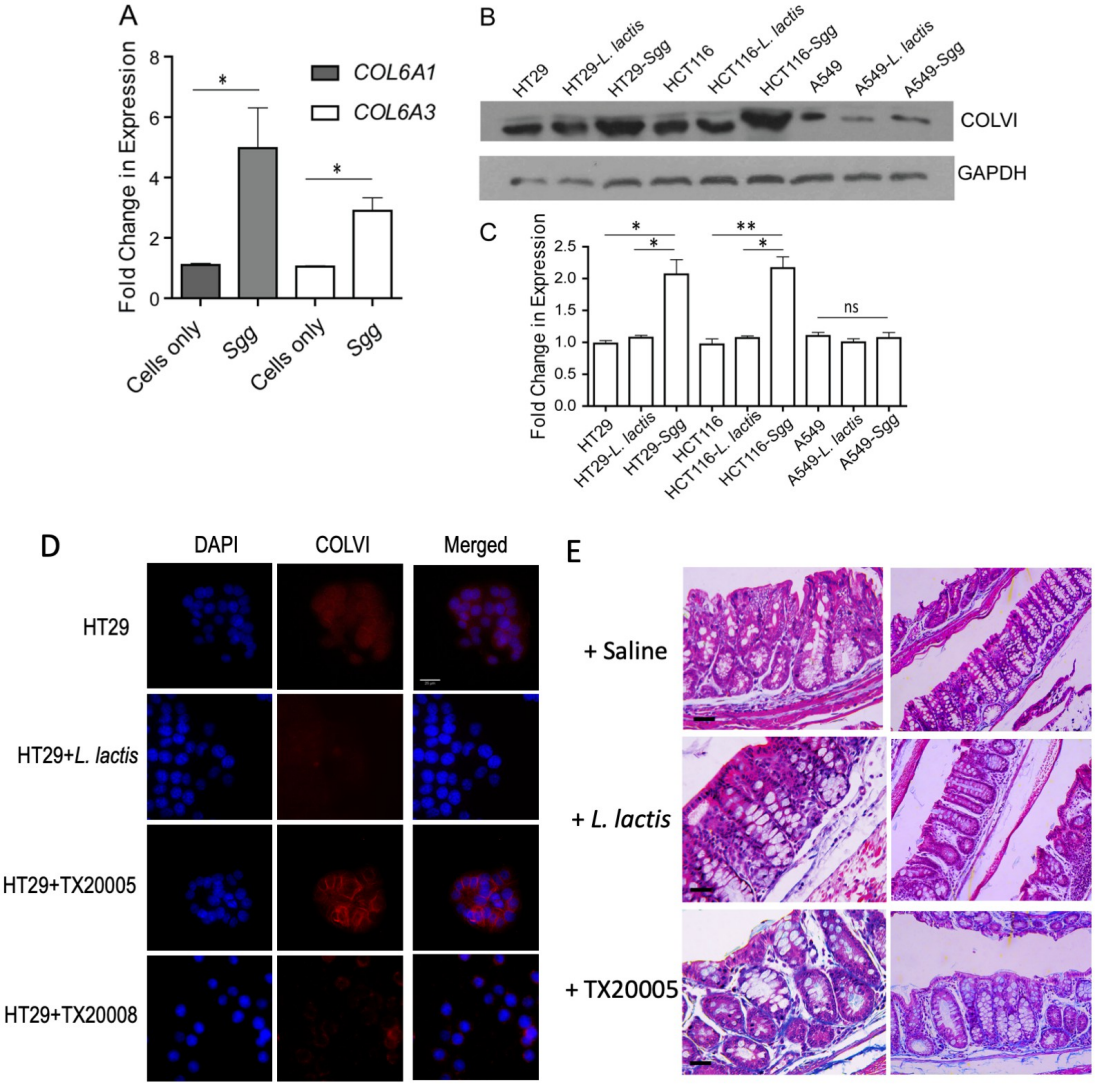
*Sgg* upregulates collagen expression in cultured cells. **A**. HT29 cells were co-cultured with *Sgg* strain TX20005 or media only for 6 hours. RNA was extracted and analyzed by RT-qPCR. CT values were first normalized to GAPDH then to cells cultured in media only and then converted to fold changes. **B** and **C**. HT29, HCT116 and A549 cells were co-cultured with *Sgg* TX20005, *L*. *lactis*, or media only for 12 hours. Cell lysates were subject to western blot with anti-ColVI antibody. Band intensity was quantified using Image J and normalized to GAPDH. Data presented is mean ± SEM from three independent experiments. **D**. HT29 and HCT116 were co-cultured with *Sgg* TX20005, TX20008, *L*. *lactis*, or media only for 12 hours. Cells were washed, fixed, incubated with anti-ColVI antibody and counterstained with DAPI. Representative images are shown. Scale bars represent 25μm. **E**. Colon sections from mice orally gavaged with *Sgg* TX20005, *L*. *lactis* or saline were stained with Trichrome stains. Collagen is stained blue. Statistical analysis in **A** and **C** was done using unpaired, two-tailed *t* test. *, *p* < 0.05; **, *p* < 0.01.

*Sgg* was previously shown to stimulate the proliferation of certain human CRC cells including HT29 and HCT116 cells [10,14]. To investigate the changes in CRC cells induced by *Sgg*, we performed mass spectrometry-based label-free global proteome profiling of whole cell lysates prepared from HT29 cells cultured alone or in the presence of *Sgg* strain TX20005 (S1 Table). Strikingly, the level of several types of collagens was increased in cells co-cultured with TX20005, with type VI collagen (ColVI) showing the highest relative abundance (S2 Table). The increased expression of ColVI was further confirmed at the transcription and protein level. In RT-qPCR, both ColVI α1 chain (COL6A1) and α3 chain (COL6A3) were significantly increased in the presence of TX20005 compared to cells cultured in media only (Fig 1A). In western blot, ColVI level was significantly increased in HT29 and HCT116 cells co-cultured with TX20005, compared to cells co-cultured with *Lactococcus lactis*, a non-pathogenic negative bacterial control, or in media only (Fig 1B and 1C). Previous studies showed that *Sgg* stimulates the proliferation of HT29 and HCT116 cells, but had no effect on A549 cells, a human lung cancer cell line [10]. No significant changes in ColVI were observed in A549 cells cultured in the presence of TX20005 when compared to cells cultured in the presence of *L*. *lactis* or in media only (Fig 1B and 1C). Using immunofluorescence (IF) microscopy, we further validated that TX20005 upregulated ColVI (Fig 1D and S1 Fig). Upregulation of type I collagen (ColI) by TX20005 was also confirmed by using IF (S2 Fig).

We further tested the effect of *Sgg* strain TX20008 on ColVI. TX20008 was previously shown to be unable to promote the proliferation of HT29 or HCT116 cells (designated as NP-*Sgg* for non-proliferation-promoting *Sgg*) [12]. IF microscopy data showed that TX20008 had no effect on ColVI (Fig 1D). This was further confirmed by RT-qPCR which showed no increase of COL6A1 in TX20008 treated HT29 cells (S3 Fig).

We next examined the effect of *Sgg* TX20005 on collagen expression *in vivo* using colon sections from mice orally gavaged with TX20005, *L*. *lactis* or saline. Sections were stained with Masson’s Trichrome stain which stains collagen blue [32]. The results showed that colon sections from mice gavaged with TX20005 had more intense blue staining compared to sections from mice gavaged with *L*. *lactis* or saline (Fig 1E), indicating elevated level of collagen following exposure to *Sgg* TX20005. IF staining of the colon sections with an anti-ColVI antibody also showed more intense staining of ColVI in the colonic crypts from TX20005-gavaged mice compared to control mice (S4 Fig). Taken together, these results indicate that exposure to *Sgg* TX20005 results in increased level of collagen in *in vitro* cultured cells and in the intestinal mucosa *in vivo*.

### Collagen is required for *Sgg* to stimulate human CRC cell proliferation

**Fig 2:**
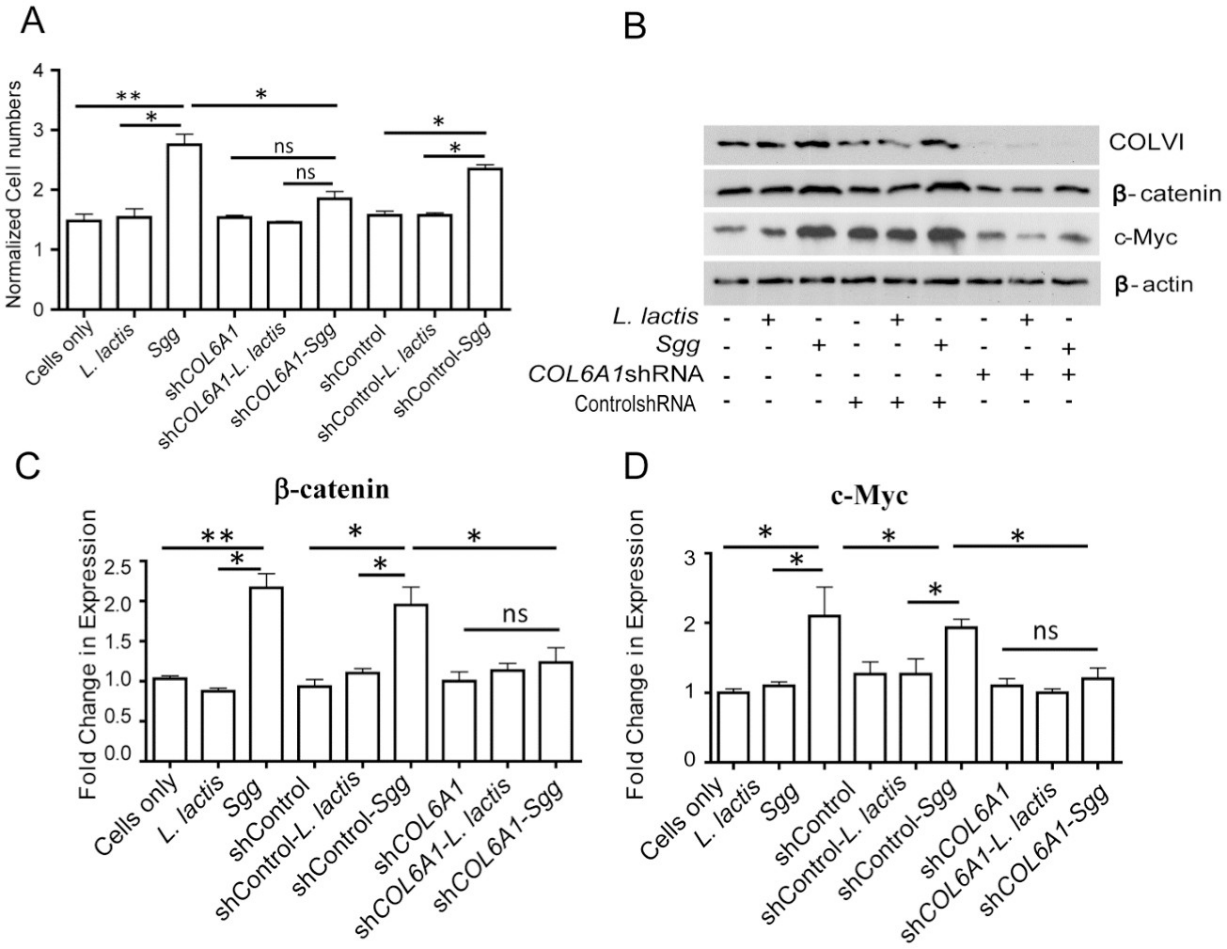
*Sgg* promotes cell proliferation in a ColVI-dependent manner. **A**. Knockdown of COL6A1 abolished the effect of *Sgg* on cell proliferation. Untransfected HT29 cells, COL6A1 stable knockdown HT29 cells or HT29 cells transfected with a control shRNA were incubated with media only, *L*. *lactis* or *Sgg* TX20005 for 24 hours. Cell proliferation assays were performed by counting viable cells as described in the Materials and Methods section. **B-D**. Cells were incubated in media only, *Sgg* TX20005 or *L*. *lactis* for 12 hours as described in the Materials and Methods section. Total cell lysates were subject to western blot assays to compare ColVI, β-catenin, and c-Myc protein levels. Representative images are shown (**B**). Band intensity was quantified using Image J, normalized to β-actin first and then to the media only control (**C-D**). Data are presented as the mean ± SEM. Each experiment was repeated at least three times. Unpaired, two-tailed *t* test was used for statistical analysis. *, *p* < 0.05; **, *p* < 0.01.

Collagen has been shown to mediate the proliferation of cancer cells [33–36]. We investigated the role of collagen in *Sgg*-induced CRC cell proliferation. HT29 COL6A1 and COL6A3 stable knockdown cells were generated. The ability of TX20005 to stimulate the proliferation of either COL6A1 (Fig 2A) or COL6A3 (S5A Fig) knockdown cells was significantly reduced compared to that in untransfected cells or cells transfected with control shRNA. We confirmed that COL6A1 (Fig 2B) or COL6A3 (S5B Fig) knockdowns reduced the level of ColVI in the cells. *Sgg* was shown to upregulate β-catenin and c-Myc and β-catenin is required for *Sgg* to stimulate cell proliferation [10]. Knockdown of COL6A1 (Fig 2B–2D) or COL6A3 (S5B–S5D Fig) suppressed *Sgg*-induced upregulation of β-catenin or c-Myc, suggesting that ColVI acts upstream of β-catenin in the signaling cascade that leads to *Sgg*-induced cell proliferation. We note that a small insignificant increase was still observed in cell proliferation, β-catenin and c-Myc in *Sgg*-treated ColVI knockdown cells. This could be due to the knockdown being incomplete since a low level of ColVI was seen in the knockdown cells (Fig 2B and S2B Fig) and/or redundancy of other pathways.

In addition to ColVI, we also carried out knockdown of ColI using siRNA specific for the α1 chain of ColI (COL1A1). COL1A1 knockdown abolished the ability of *Sgg* to stimulate cell proliferation (S6 Fig), suggesting that multiple ECM components are involved in *Sgg*-induced cell proliferation.

### Collagen is involved in *Sgg* adherence to CRC cells but is not a major determining factor

**Fig 3:**
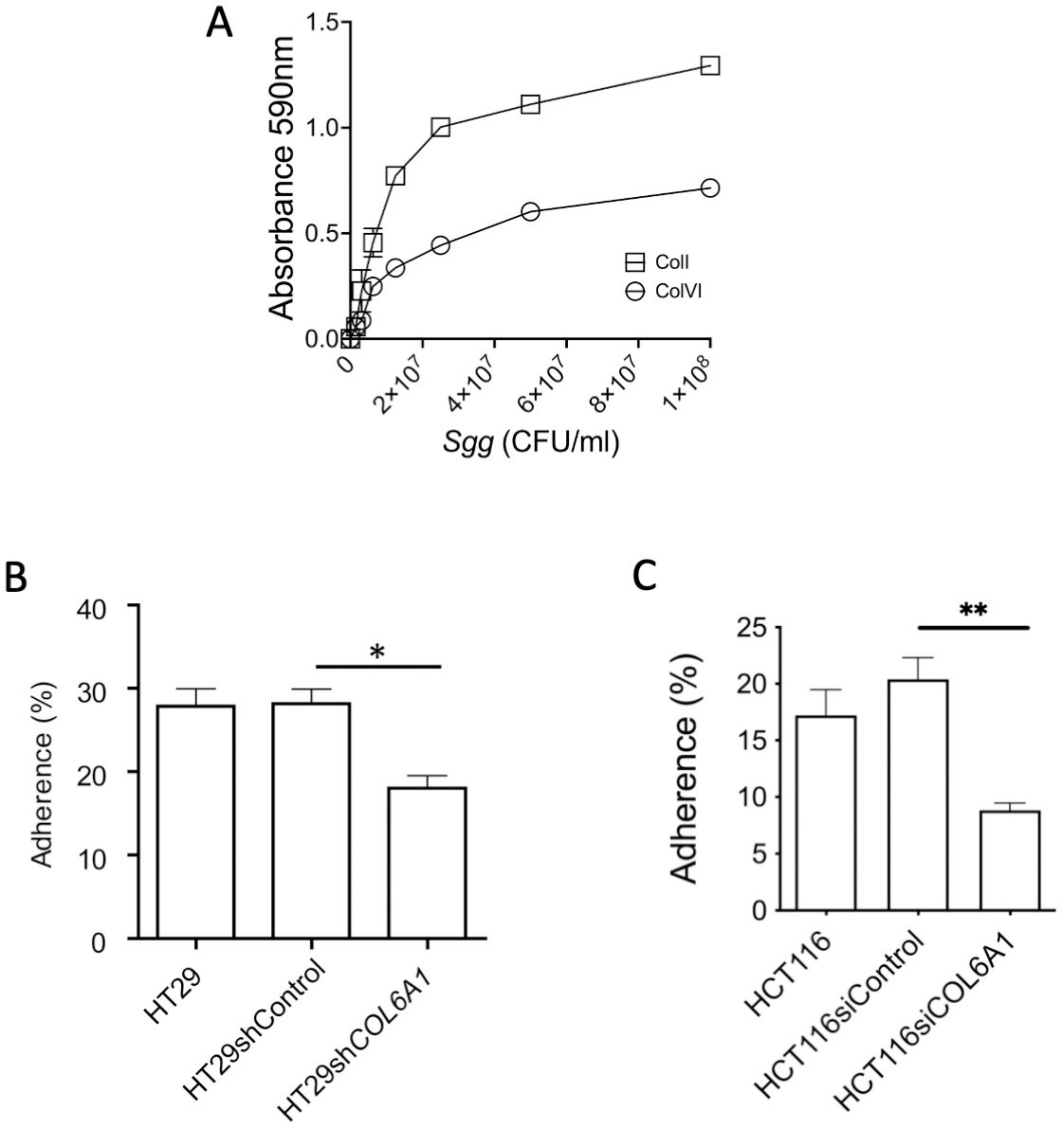
Collagen knockdown reduced the adherence of *Sgg*. **A.** *Sgg* binds ColI and ColVI in a dose-dependent manner. Serially diluted TX20005 in PBS was added to immobilized human ColI and ColVI in 96-well plates, as described in the Materials and Methods section. Bound bacteria were detected using crystal violet. The experiments were repeated three times. Mean ± SD is presented. **B** and **C**. *Sgg* adherence to ColVI knockdown cells. HT29, HT29 transfected with a control shRNA or COL6A1 stable knockdown HT29 cells (**B**), HCT116, HCT116 transfected with a control siRNA or COL6A1 siRNA (**C**) were incubated with TX20005 (MOI = 10) as described in the Materials and Methods section. Adherence was calculated as the percentage of adhered bacteria vs. total bacterial added and combined from at least three independent experiments. Mean ± SEM is presented. Unpaired, two-tailed *t* test was used for statistical analysis. *, *p* < 0.05.

Several *Sgg* strains including TX20005 were previously shown to bind collagen type I, IV and V [37–39]. We confirmed that TX20005 also bind ColVI in a dose-dependent manner, although not as strong as to ColI (Fig 3A). We investigated if knockdown of ColVI affected the adherence of TX20005 to CRC cells and found that adherence to COL6A1 knockdown HT29 (Fig 3B) and HCT116 (Fig 3C) cells was reduced by ~40% and ~50%, respectively. These results suggest that binding to ColVI contributes to *Sgg* adherence, however interaction with other factors on the host cell surface are equally or maybe more important for *Sgg* to adhere to these cells.

Previous work showed that *Sgg* strains exhibit different adherence capacity to HT29 cells, with TX20030 and TX20031 (PP-*Sgg* strains) adhere better than TX20005, whereas TX20008 and ATCC43143 (NP-*Sgg* strains) significantly poorer than TX20005 [12]. We tested the ability of these strains to bind ColI and ColVI. The results showed that all strains exhibited similar binding to ColI or ColVI (S7 Fig), consistent with previous data that the collagen adhesin Acb is widely expressed among *Sgg* strains [38]. *Sgg* was shown to bind other components of the ECM in addition to ColI and ColVI. We further tested the ability of these *Sgg* strains to bind the ECM produced by HT29 and HCT116 cells. Decellularized matrix (dc-matrix) was prepared from HT29 and HCT116 cells transfected by a control siRNA or COL6A1 siRNA. ColVI knockdown was confirmed by western blot (S8 Fig). We also confirmed that no intact cells remained after the decellularization procedure by staining the samples with DAPI (S9 Fig). The ability of the *Sgg* strains to bind to the dc-matrices were then examined. The results showed that there is no significant difference between the strains in their ability to bind the dc-matrices. However, binding to dc-matrices prepared from COL6A1 knockdown HT29 or HCT116 cells was significantly reduced, as expected (S10A and S10B Fig). Depletion of ColVI may affect other components of the ECM [40–42], hence the reduced binding to dc-matrices from COL6A1 knockdown cells may also be a consequence of changes of other ECM components caused by the knockdown.

Taken together, these results suggest that the ability to bind collagen or the ECM is not a major determining factor for *Sgg* adherence to CRC cells. This is likely due to the possibility that most of the ECM molecules are deposited on the basal side of the cells not readily accessible to *Sgg* under the test conditions. Interaction with other factors on the host cell surface is likely to be more important for *Sgg* to adhere to these host cells. The results also suggest that the ability to bind collagen and the ECM is not correlated to the ability of *Sgg* to induce CRC cell proliferation.

### Dc-matrix derived from *Sgg*-treated cells alone is sufficient to promote cancer cell proliferation

**Fig 4:**
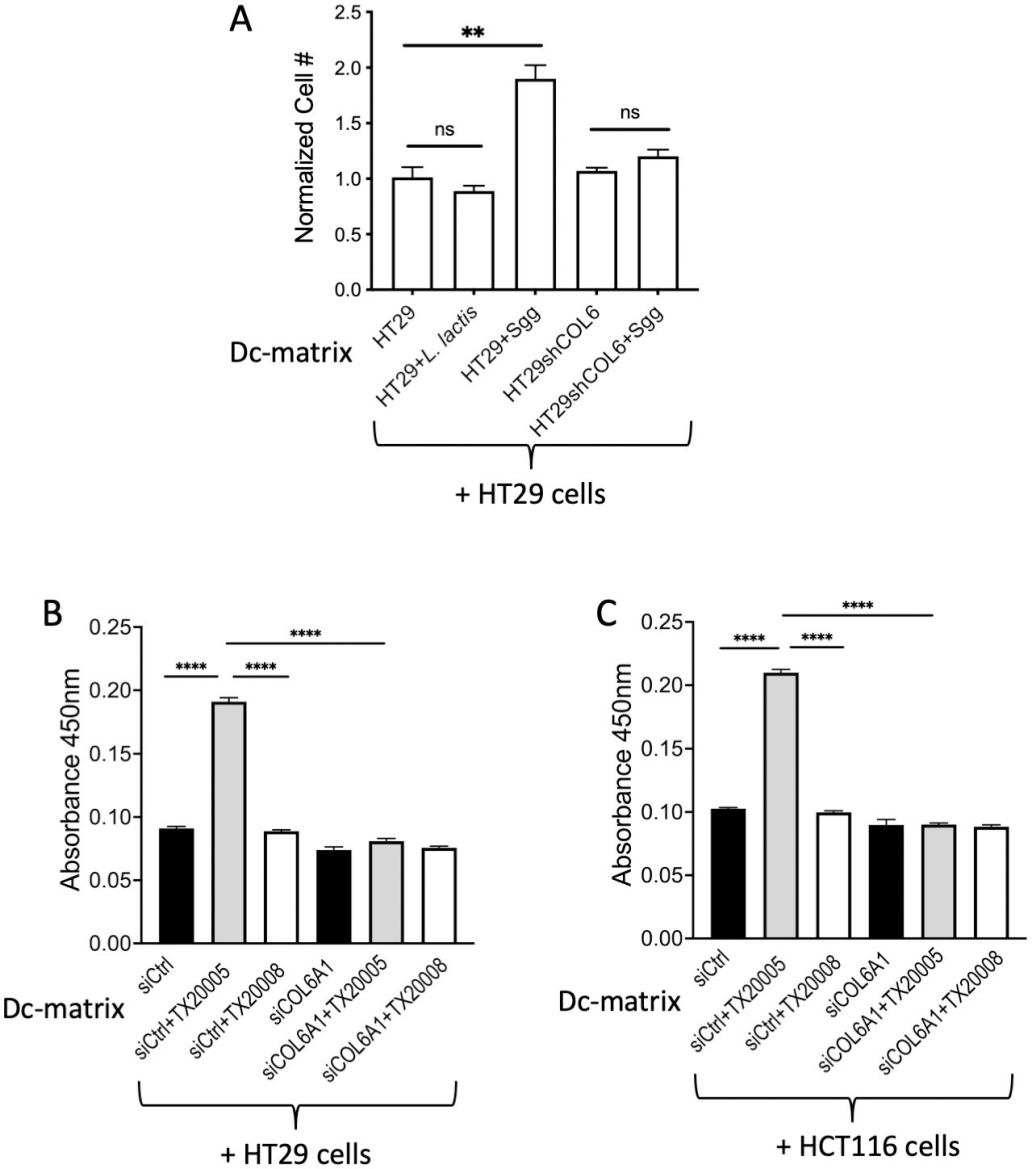
Decellularized matrix (dc-matrix) derived from PP-*Sgg*-treated cells is sufficient to stimulate cell proliferation. **A.** HT29 cells and COL6A1 stable knockdown HT29 cells were co-cultured with *Sgg* strain TX20005, *L*. *lactis* or media only for 12 hours. The wells were incubated with antibiotics to eliminate bacteria followed by washing. Cells were then stripped away from the underlying matrix as described in the Materials and Methods section. HT29 cells (~ 1 x 10^4^) that had not been previously exposed to *Sgg* were seeded on the indicated dc-matrices and incubated for 24 hours. Viable cells were counted. **B** and **C**. HT29 (**B**) and HCT116 (**C**) cells were transiently transfected with control siRNA or COL6A1 siRNA and then co-cultured with *Sgg* TX20005, TX20008 or media only. Dc-matrices were then prepared. Fresh HT29 and HCT116 cells that had not been previously exposed to *Sgg* were seeded onto the indicated dc-matrices and incubated for 24 hours. Cell proliferation was examined using the CCK-8 assay. Each experiment was repeated at least three times. Data is presented as the mean ± SEM. Statistical analysis was done using unpaired, two-tailed *t* test. **, *p* < 0.01; ****, *p* < 0.0001.

It is known that increased collagen deposition leads to matrix-induced cell proliferation [35,36]. Therefore, it is possible that collagen contributes to *Sgg*-stimulated cell proliferation in this fashion. To investigate this possibility, dc-matrix was prepared from HT29 cells cultured in media only or in the presence of bacteria. We confirmed that no live bacteria were present in the dc-matrix by incubating dc-matrix in antibiotics-free media for 24 hours. No bacterial growth was observed. HT29 cells were then seeded onto the various dc-matrices and incubated in antibiotics-containing media for 24 hours. The dc-matrix prepared from TX20005-treated cells stimulated cell proliferation significantly better than the dc-matrix from HT29 cells alone or *L*. *lactis*-treated cells (Fig 4A). Furthermore, dc-matrices were also prepared from COL6A1 stable knockdown HT29 cells that had been incubated with or without TX20005. The dc-matrix from *Sgg*-treated COL6A1 HT29 cells showed no significant difference in the ability to stimulate cell proliferation compared to the dc-matrix prepared from COL6A1 HT29 cells alone (Fig 4A), suggesting that ColVI is important for the effect of the ECM on cell proliferation.

To further validate the results, HT29 and HCT116 cells were transiently transfected with a control siRNA or COL6A1 siRNA and then cultured in media only, or in the presence of *Sgg* TX20005 or TX20008 (as a negative control). Dc-matrices were then prepared, onto which fresh HT29 and HCT116 cells were seeded (Fig 4B and 4C). CCK-8 assay was then used to determine cell proliferation as an independent method from the enumeration of viable cell method used in Fig 4A. Similar results were obtained. In both cell lines, dc-matrices from TX20005-treated cells stimulated cell proliferation in a ColVI-dependent manner. In contrast, dc-matrices from TX20008-treated cells showed no difference compared to that from untreated cells in either control siRNA transfected or COL6A1 siRNA transfected cells (Fig 4B and 4C).

Next, we investigated dc-matrices prepared from HT29 and HCT116 cells cultured in the presence of other PP-*Sgg* strains TX20030 and TX20031 and NP-*Sgg* strain ATCC43143 [12]. Interestingly, co-culture with TX20030 and TX20031 yielded dc-matrices that were effective at stimulating cell proliferation and that the effect was dependent on ColVI, similar to TX20005 (S10A and S10B Fig). On the other hand, dc-matrices from ATCC43143-treated cells showed no difference to those from untreated cells regardless of ColVI knockdown, similar to TX20008.

Taken together, these results suggested that the ECM from PP-*Sgg* treated cells stimulates cell proliferation in a manner that does not require live *Sgg* but depends on ColVI, whereas the ECM from *L*. *lactis* or NP-*Sgg* treated cells were ineffective.

### ColVI is required for *Sgg* to promote tumor growth *in vivo*

**Fig 5:**
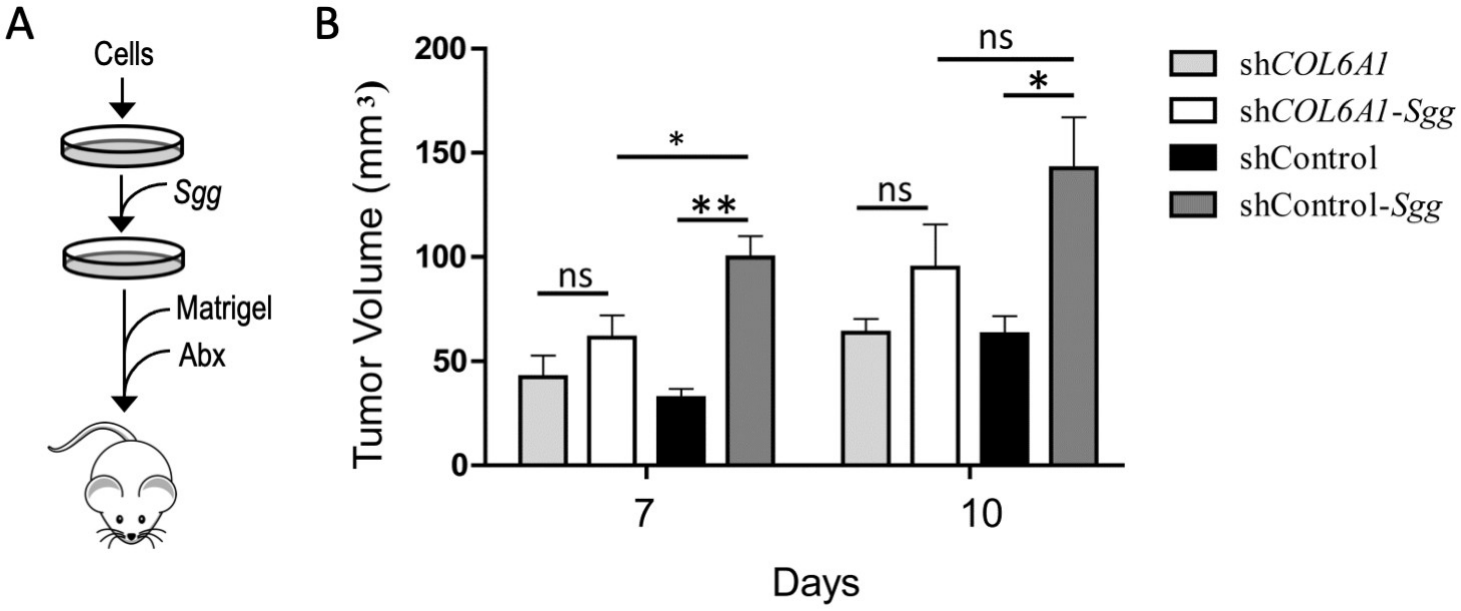
Collagen knockdown impairs the ability of *Sgg* to promote tumor growth *in vivo*. **A.** A schematic diagram of the animal procedure. 1 x 10^6^ HT29shCOL6A1 or HT29shcontrol cells were treated with *Sgg* TX20005 or no bacteria as in cell proliferation assays, mixed with Matrigel and injected into the dorsal flap of nude mice (n = 5/group). Mice were then treated with antibiotics to prevent bacterial infection and monitored. **B.** Tumor size was measured at the indicated time point with a digital caliper. Data is presented as the mean ± SEM. Statistical analysis was done using unpaired, two-tailed *t* test. *, *p* < 0.05; **, *p* < 0.01.

To determine the importance of ColVI in *Sgg*-induced tumor growth *in vivo*, shCOL6A1 knockdown cells and cells transfected with control shRNA were cultured in the absence or presence of *Sgg* TX20005 and then injected into nude mice (Fig 5A). For cells transfected with the control shRNA, TX20005-treatment resulted in significantly larger tumors at day 7 and 10 post injection compared to untreated cells (Fig 5B). In shCOL6A1 knockdown cells, TX20005-treatment led to slightly larger tumors, however the difference was insignificant compared to untreated knockdown cells. As mentioned earlier, the small increase in *Sgg*-treated COL6A1 knockdown cells could be due to incomplete knockdown and/or redundancy of the pathways. We note that in order to prevent infection caused by *Sgg*, mice were administered with antibiotics following injection of cells to eliminate *Sgg*. Therefore, the effect of *Sgg* on tumor growth was more pronounced at day 7 than that at day 10. Altogether these results suggest that ColVI is important for *Sgg* to promote tumor growth *in vivo*.

## Discussion

Previous studies showed that certain *Sgg* strains (PP-*Sgg*) actively stimulate CRC cell proliferation and promote tumor growth, however the molecular mechanisms underlying these phenotypes are not well understood. Here we provide evidence that the prototypic PP-*Sgg* strain TX20005 upregulates collagen expression *in vitro* and *in vivo*. Furthermore, we demonstrate that the ECM is a direct mediator of *Sgg*-induced cell proliferation and is important for *Sgg*-induced tumor growth *in vivo*. To the best of our knowledge, this is the first report that describes a dynamic bidirectional interplay between a gut microbe and the ECM in the context of microbial contribution to cancer.

**Fig 6:**
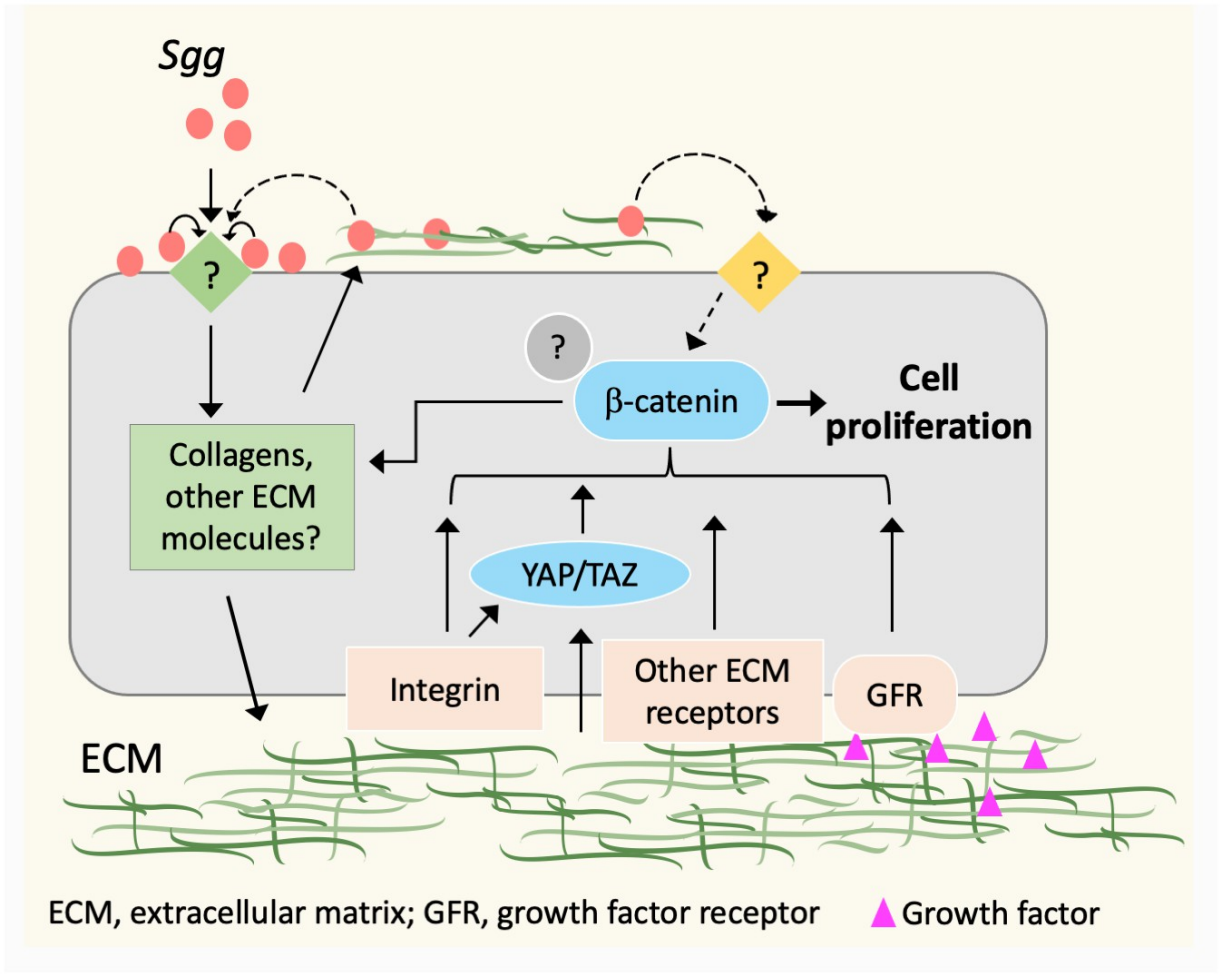
A schematic diagram of *Sgg*-induced ECM changes contributing to increased cell proliferation. *Sgg* upregulates the expression of collagens via unknown receptors, leading to increased deposition of collagens in the ECM. This results in enhanced ECM-receptor signaling (integrins and other ECM receptors) which then regulate β-catenin. Increased collagen deposition may also alter the stiffness of the matrix or trap more growth factors. Mechanical cues can be sensed by integrins or mechanotransducers YAP/TAZ. Increased collagen deposition also allows more *Sgg* to adhere via the collagen adhesin Acb/Pil1 or other MSCRAMMs, enabling *Sgg* factors to interact with host cell receptors in proximity.

Our data indicates that ColVI and ColI are important for *Sgg*-induced CRC cell proliferation. ColVI has a unique supramolecular structure among the members of the collagen family. Its beaded microfilament structure enables it to bind to other components of the ECM such as ColI and ColII and acts as a bridging molecule [40–42]. Thus, depletion of ColVI may affect the overall organization and composition of the ECM [17,43,44]. This, combined with the result that ColI is also required for *Sgg* to stimulate CRC cell proliferation, indicates that multiple ECM components are involved. A working model for how the ECM contributes to *Sgg*-induced CRC cell proliferation is proposed (Fig 6).

There are several possible ways that the ECM can contribute to *Sgg*-induced CRC cell proliferation. One is through ECM-receptor signaling. Upregulation of collagen by *Sgg* can lead to increased deposition of collagen into the matrix, which results in enhanced signaling from collagen receptors such as integrins α_1_β_1_ and α_2_β_1_ [45]. Increased collagen deposition may also trap more other matrix molecules including fibronectin [46], which signals through receptors such as integrin α_5_β_1_ [47]. Previous work showed that *Sgg* stimulates CRC cell proliferation in a β-catenin-dependent manner, however the signaling events upstream of β-catenin were unknown [10]. Multiple studies have shown that integrin regulates β-catenin signaling in response to ECM stimuli. For example, ColI [48] and fibronectin [49] positively regulates β-catenin signaling via integrin β_1_. Regulation of β-catenin by integrin-linked kinase has also been reported [50,51]. Thus, *Sgg*-induced ECM changes can lead to increased cell proliferation via integrin signaling to β-catenin. Other ECM receptors may also respond to *Sgg*-induced ECM changes to regulate cell proliferation. Second, increased deposition of collagen into the matrix may alter the mechanical property of the matrix. A stiffer matrix can promote cell proliferation by mechanotransduction [52]. In addition to integrins, the Hippo pathway transcription factors YAP (Yes-associated protein) and TAZ (transcriptional coactivator with PDZ-binding motif) are major mechanotransducers that relay mechanical cues from the ECM to regulate cell proliferation [52–55]. YAP/TAZ are known to regulate the Wnt/β-catenin signaling pathway at multiple levels [56–58]. Third, the ECM serves as a “reservoir” for growth factors which can be released to activate growth factor receptors [16]. Thus, *Sgg*-induced ECM changes may provide multiple signals to trigger complex cellular signaling events that converge at β-catenin or transcription coactivators that complex with β-catenin, resulting in increased cell proliferation. In addition, β-catenin signaling has been shown to regulate ECM expression [59], which can lead to a potential positive feedback loop that further amplifies the effect of the *Sgg*-ECM interplay. We note that ColVI is special in the sense that a C-terminal soluble cleavage product of COL6A3 chain (endotrophin (ETP)) was found to augment breast tumor growth [60]. However, our results from dc-matrix and ColI knockdown experiments speak against a role of ETP in *Sgg*-induced CRC cell proliferation, however this possibility cannot be completely excluded. Delineating the signaling pathways downstream of the ECM will shed light on how *Sgg* modification of the ECM contributes to the pro-proliferative effect of *Sgg*.

The ECM may also affect the pro-proliferative capacity of *Sgg* in an indirect way by trapping more *Sgg* bacteria close to the cell surface (Fig 6, indicated by the dashed lines). While most ECM molecules are deposited on the basal side of the cells, a small proportion may be exposed on the surface of CRC cells accessible to *Sgg*. This can enhance *Sgg* adherence via the collagen adhesin Acb or other MSCRAMMs (Microbial Surface Component Recognizing Adhesive Matrix Molecules) of *Sgg* [38], as supported by our adherence results. Previous studies have shown that secreted PP-*Sgg* factors are sufficient to stimulate the proliferation of certain CRC cells [14,15]. Increased adherence allows relevant secreted factors to interact with host cell receptors in proximity and at relatively high local concentrations to regulate ECM expression. Additionally, ECM-mediated adherence may enable certain *Sgg* secreted factors to activate other unknown receptors to directly influence β-catenin signaling, thereby contributing to cell proliferation.

Components of the ECM such as collagen and fibronectin are commonly targeted by bacterial pathogens to facilitate adherence to and invasion of host cells and colonization of host tissues [61–63]. *Sgg* expresses a collagen adhesin Acb at the tip of the Pil1 pilus (also known as Gallo2179) [38,39]. The adhesin is widely distributed among *Sgg* strains [38] and is important for virulence in experimental endocarditis [39]. Here we found that despite their differences in impacting CRC cell proliferation [12], the *Sgg* strains investigated in this study exhibit similar binding to ColI, ColVI and the dc-matrix, consistent with previous results [38]. Considering that these *Sgg* strains are clinical isolates from endocarditis patients [37] and the importance of collagen binding in experimental endocarditis [39,64–67], this result is not surprising. Our results also suggest that while collagen binding contributes to *Sgg* adherence, it is likely not a key determinant for *Sgg* adherence to the CRC cell lines under the condition tested in this study. Interaction with unknown host cell receptors likely plays a more important role. On the other hand, tissue injury in the colonic epithelium or the microenvironment around tumors may make the ECM more accessible to *Sgg*. Under these circumstances, binding to collagen and other ECM molecules may become more important for *Sgg* to attach to and invade the colonic or tumor tissues.

In contrast to a large body of work investigating the binding interactions between microbial adhesins and ECM molecules, knowledge of microbial modulation of the ECM is scant. Here, our results show that *Sgg* not only binds collagen but also actively regulate their expression. The *Sgg*-modified ECM in turn mediates CRC cell proliferation and contributes to *Sgg* adherence. Thus, this study reveals a novel bidirectional interplay between the pathogen and the ECM. How *Sgg* upregulates collagen expression is unknown. ECM expression can be regulated by multiple pathways. Future work to identify the *Sgg* factors and the host cell pathways targeted by *Sgg* for regulating ECM expression will be critical.

In this study, we focused on the effect of *Sgg* on CRC cells. Fibroblasts are major producers of ECM molecules *in vivo*. Tumor-associated macrophages were also shown to regulate the synthesis and assembly of collagenous matrix [68]. Currently the effect of *Sgg* on fibroblasts and macrophages, which are major components of the tumor microenvironment, is unknown. In addition, whether *Sgg* affects ECM expression in cardiovascular cells, which is relevant to *Sgg* IE, is also unclear. Elucidating the influence of *Sgg* on ECM expression in different cell types will be important for understanding the contribution of *Sgg* modulation of ECM to the pathogenic potential of *Sgg* in different settings. From the perspective of microbial association with CRC, it is well appreciated that specific gut microbes or microbial communities play important roles in influencing the development of CRC. The molecular mechanisms used by other microbes to drive the development of CRC can be loosely grouped into the following categories: 1) producing genotoxins that directly induce DNA damage in colonic epithelial cells, 2) modulating host immune responses to generate a microenvironment favorable for CRC, and 3) shifting host metabolism to support tumor growth [69–77]. Results from this study suggest a novel strategy by which microbes influence CRC development—via the ECM.

In conclusion, this study provides the first experimental evidence for *Sgg* modulation of the ECM and a direct role of the ECM in *Sgg*-induced cell proliferation and tumor growth. The results presented here highlight a novel dynamic two-way interplay between *Sgg* and the ECM that have important implications to understanding *Sgg* contribution to CRC. Going forward, studies to dissect the signaling pathways upstream and downstream of *Sgg*-induced ECM will be critical.

## Materials and methods

### Ethics statement

Animal studies were performed in accordance with protocols (IACUC#2017-0420-IBT) approved by the Institutional Animal Care and Use Committee at the Texas A&M Health Science Center, Institute of Biosciences and Technology. The Texas A&M University Health Science Center—Institute of Biosciences and Technology is registered with the Office of Laboratory Animal Welfare per Assurance A4012-01. It is guided by the PHS Policy on Human Care and Use of Laboratory Animals (Policy), as well as all applicable provisions of the Animal Welfare Act. Mice were euthanized by CO_2_ inhalation followed by cervical dislocation.

### Bacteria and cell culture conditions

*Sgg* strains and *Lactococcus lactis* MG1363 were cultured as described previously [10]. Human colon cancer cell line HT29 and HCT116 were cultured in DMEM/F-12 HEPES (GIBCO, USA) supplemented with 10% fetal bovine serum (FBS) (GIBCO, USA). Human lung carcinoma cell line A549 was maintained in F12-K media supplemented with 10% FBS. All the cells were cultured in a humidified incubation chamber at 37°C with 5% CO_2_.

### Preparation of dc-matrix

Cells were decellularized following a protocol described previously [78]. Briefly, cells were washed thrice with ice-cold sterile PBS containing a cocktail of protease inhibitors (GenDEPOT). The cells were then incubated in a PBS solution containing 0.25% Triton X and 0.25% sodium-deoxycholate for 5 minutes, followed by gentle washing in PBS thrice and incubation with 100 mg/mL RNAse A (Roche) and 10 IU/mL DNAse (Sigma) for 30 minutes. The samples were then washed thrice with ice-cold PBS.

### Cell proliferation assays

Co-culture with *Sgg* was performed as described previously [10]. Briefly, cells (~1x10^4^ cells/well) were cultured in the presence of stationary phase bacteria at a multiplicity of infection (MOI) of 1 or media only for 24 hours. Trimethoprim was added at 50 μg/ml final concentration after 6 hours of co-culture to prevent media acidification due to bacterial growth. To examine cell proliferation on dc-matrix, cells were seeded onto dc-matrix prepared from different treatment conditions and incubated in tissue culture media containing penicillin and streptomycin for 24 hours. Two methods were used to examine cell proliferation. 1) Counting viable cells as described previously [10]. Cells were detached by trypsin treatment and counted in a Cellometer Mini automated cell counter (Nexcelome Biosciences, Lawrence, MA). 2) Cell Counting Kit 8 (CCK-8) following the instructions from the supplier (Apex Bio).

### Collagen knockdown

To generate stable knockdown cells, lentiviral plasmids containing COL6A1 or COL6A3 short hairpin RNA (shRNA) (Sigma-Aldrich, TRCN0000116959 and TRCN0000003622), or MISSION pLKO.1-puro Non-Mammalian shRNA Control (Sigma-Aldrich, SHC002) were first transfected into HEK293T cells to produce lentiviral particles. HT29 cells were then infected with the respective lentiviral particles and selected with puromycin (1μg/ml). Gene knockdown was confirmed by western blot assays. Transient knockdown of COL1A1 and COL6A1 was carried out using specific siRNA for COLA1 and COL6A1 (ThermoFisher) following the ThermoFisher Silencer Select siRNA protocol 2013. Western blot was performed to confirm the knockdown.

### Quantitative reverse transcription PCR (RT-qPCR)

HT29 cells were co-cultured with *Sgg* for 6 hrs. Total RNA was extracted from co-cultured cells using the RNeasy Kit (QIAGEN). cDNA was generated by using the Transcriptor First Strand cDNA Synthesis Kit (Roche). qPCR was performed using FastStart SYBR green master mix (Roche) in a Viia 7 Real Time PCR System (Applied Biosystems). The following cycle conditions were used: 95°C for 10 minutes followed by 40 cycles at 95°C for 30 seconds and 60°C for 1 minute. CT values were first normalized to GAPDH then to cells cultured in media only.

### Western blot assays

This was performed as described previously [10]. Briefly, cells were cultured in the appropriate medium in the presence or absence of bacteria for 12 hours, washed and lysed. Total cell lysates were subjected to SDS-gel electrophoresis and western blot. Rabbit polyclonal antibodies against ColVI (1:500, Abcam), β-catenin (1:4000, Cell Signaling Technology (CST)), c-Myc (1:3000, Abcam), and β-actin (1:5000, CST) were used. Horse radish peroxidase (HRP)-conjugated anti-rabbit IgG (CST) was used as the secondary antibody. Signals were detected using HyGLO, chemiluminescent HRP (Denville, Mteuchen, NJ). Band intensity was quantified using Image J.

### Bacterial binding assay

This was carried out using the crystal violet method following a procedure described previously [79] with slight modifications. Briefly, 96-well plates were coated with purified native human ColI (abcam), ColVI (abcam), or BSA at 100 ng/well. Wells were then washed and blocked with PBS containing 3% BSA. Indicated concentrations of bacteria resuspended in sterile PBS were added to each well and incubated for 1 hour at 37°C in a humidified chamber with 5% CO_2_. Wells were washed thrice with PBS, fixed with 100% ice-cold methanol for 10 minutes, washed thrice more in PBS, and incubated in 0.5% crystal violet for 5 minutes. After washing, 100 μl of room temperature methanol was used to solubilize the crystal violet stain and absorbance at 590nm was read to determine bound bacteria.

### Adherence assay

This was performed as described previously [10]. Briefly, cells were incubated with or without bacteria at an MOI of 10 for 1 hour. The wells were washed three times with sterile PBS to remove unbound bacteria. To determine the number of bound bacteria, cells were lysed with sterile PBS containing 0.025% Triton X-100 and dilution plated. Adherence was expressed as a percentage of total bacteria added.

### Animal experiment

The xenograft experiment was performed as described previously [10]. Tumor diameters were measured with a digital caliper, and tumor volume calculated using the formula: Volume = (d1xd1xd2)/ 2, with d1 being the larger dimension.

### Immunofluorescence

#### 1) Colon sections

Colon sections were from previous animal studies using an AOM-induced CRC model in A/J mice [10]. Methcarn-fixed paraffin embedded colon sections were deparaffinized with xylene and rehydrated in an ethanol gradient. The slides were incubated in a citrate buffer at 95°C for 15 minutes, cooled to room temperature (RT), rinsed with PBS and incubated in a blocking buffer (PBS containing 1% Saponin and 20% BSA) for 30 minutes. The slides were then incubated with rabbit anti-ColVI (1:200, Abcam) at 4°C overnight, washed with PBS, and incubated with donkey-anti-rabbit Alexa 594 for 1 hour at RT. The slides were washed again, stained with DAPI, mounted and examined in a DeltaVision Elite microscope (GE Healthcare).

#### 2) Cultured cells

Cells were seeded onto an 8-chambered slide and cultured under various conditions. Cells were washed, fixed with 4% formaldehyde, and permeabilized with 0.1% Triton-X-100 for 30 minutes. Cells were then incubated in a blocking solution (PBS containing 5% donkey serum and 0.3% Triton X-100) for 1 hour. The slides were then incubated with anti-ColVI or anti-ColI antibodies (1:100) at 4°C overnight, washed with PBS, and incubated with donkey-anti-rabbit Alexa 594 (1:500 dilution in PBS) for 1 hour at RT. The slides were washed again, stained with DAPI, mounted and examined in a DeltaVision Elite microscope (GE Healthcare).

### Trichrome staining of colon sections

Colon sections were deparaffinized, rehydrated and stained using a Trichrome Stain Kit (Abcam, ab150686) following the protocol provided by the manufacturer.

## Supporting information

S1 Table Global proteome analysis. HT29 cells were cultured in the presence or absence of *Sgg* strain TX20005 for 24 hours. The cells were washed, lysed, and digested with Trypsin then analyzed by a nanoLC-1200 system coupled to an Orbitrap Fusion Lumos mass spectrometer. Obtained spectra were searched against the target-decoy Human RefSeq database (release 2020) in Proteome Discoverer 2.1 interface (PD 2.1, Thermo Fisher) with the Mascot algorithm (Mascot 2.4, Matrix Science). Protein inference and quantitation were performed by gpGrouper (v1.0.040) with shared peptide iBAQ area distribution (Saltzman et al 2018 PMID 30093420) then convert to iFOT. iFOT is the normalization of individual protein intensity to the total protein intensity within one experiment.
(XLSX)

S2 Table Relative abundance of several types of collagens in whole cell lysates analyzed by Mass Spectrometry ^a^. ^a^ HT29 cells were cultured in the presence or absence of *Sgg* strain TX20005 for 24 hours (3 biological replicates). The cells were washed, lysed and digested in ammonium bicarbonate buffer and trypsin. The resulting peptide mixtures were analyzed by a nanoLC-1200 system coupled to an Orbitrap Fusion Lumos mass spectrometer. Only the collagen types that show increase in all three biological replicates are listed. ^b^ Relative protein abundance is shown as the mean iFOT ± SEM. iFOT is the normalization of individual protein intensity to the total protein intensity within one experiment. ^c^ Protein not detected. The limit of detection is equivalent to iFOT = 0.0005. ^d^ Only detected in one biological replicate. ^e^ Undetermined. Statistical comparison between HT29 + *Sgg* and HT29 was not performed for these samples due to the fact that the corresponding collagen peptide chains in untreated HT29 were not detected.
(DOCX)

S1 Fig *Sgg* upregulates type VI collagen in HCT116 cells. HCT116 cells were co-cultured with TX20005 or media only for 12 hours. Cells were washed, fixed, incubated with an anti-ColVI antibodv and counterstained with DAPI.
(TIF)

S2 Fig *Sgg* upregulates type I collagen. HT29 cells were co-cultured with TX20005 or media only for 12 hours. Cells were washed. fixed. incubated with anti-Coll antibody and counterstained with DAPI.
(TIF)

S3 Fig *Sgg* TX20008 does not upregulate type VI collagen. HT29 cells were co-cultured with TX20005, TX20008 or media only for 6 hours. RNA was extracted and analyzed by RT-qPCR. CT values were first normalized to GAPDH then to cells cultured in media only and then converted to fold changes. Statistical analysis was done using unpaired, two-tailedt test. **, p<0.01.
(TIF)

S4 Fig *Sgg* upregulates type VI collagen in vivo. A/J mice were administered with 4 weekly i.p. injections of AOM, followed by treatment with ampicillin for 1 week and then weekly oral gavage of bacteria (Sgg and L. lactis, respectively) or saline for 12 weeks. Colons were harvested one week after the last bacterial gavage, swiss-rolled, fixed with meth-carn, embedded and sectioned. Colon sections were incubated with antibodies against ColVI and E- cadherin (to indicate colonic epithelial cells), followed by appropriate secondary antibodies and counterstained with DAPI.
(TIF)

S5 Fig Knockdown of COL6A3 rendered *Sgg* unable to stimulate cell proliferation. The experiments were carried out as described in Materials and Methods section. A. Cell proliferation assay. HT29 cells and COL6A3 stable knockdown HT29 cells were incubated with gg TX20005 or media only for 24 hours. Viable cells were counted using an automated cell counter. B-D. Western blot. Whole cell lysates were prepared from HT29 cells, HT29 cells with control shRNA, or COL6A3 stable knockdown HT29 cells incubated with Sgg TX20005 or L. lactis for 12 hours. Band intensity from three independent experiments were quantified and normalized to -actin. Fold change was against shCOL6A3 cells incubated in media only. Each experiment was repeated three times. Data is presented as the mean + SEM. Unpaired two tailed t test was used for pairwise comparison. *, p <0.05; **, p <0.01; ***; ns, not significant.
(TIF)

S6 Fig Knockdown of COL1A1 rendered *Sgg* unable to stimulate HT29 cell proliferation. Cell proliferation assay was carried out as described in the Materials and Methods section. HT29 cells were transfected with siRNA for COL1A1 or scrambled control siRNA and incubated for 24 hours. The cells were then incubated with L. lactis or gg TX20005 for 24 hours. Viable cells were enumerated using an automated cell counter, as described in the Materials and Methods section. Each experiment was repeated three times. Data is presented as the mean + SEM. Statistical analysis was done using unpaired, two-tailed t test. **, p < 0.01; *** p <0.001; ****, p <0.0001.
(TIF)

S7 Fig Different *Sgg* strains exhibit similar binding to COLI and COLVI. This was carried out as described in the Materials and Methods section and in the legend for Fig 3A. Briefly, serially diluted gg bacteria resuspended in PBS were added to immobilized purified human Coll or ColVI in 96-well plates. Bound bacteria were detected using crystal violet. The experiment was repeated three times. Mean + SD is presented.
(TIF)

S8 Fig Verification of ColVI transient knockdown. HT29 and HCT116 cells were transfected with a control siRNA or COL6A1 siRNA as described in the Materials and Methods section and incubated for 24 hours. Whole cell lysates were analyzed by western blot, probed with antibodies against ColVI and ß-actin, respectively.
(TIF)

S9 Fig Decellularization of HT29 cells. HT29 cells were treated with 0.25% Triton-X and 0.25% Sodium-Deoxycholate for 5 minutes and then with DNAse and RNAse for 30 minutes, as described in the Materials and Methods section. The samples were washed thrice with PBS and then fixed and stained with DAPI.
(TIF)

S10 Fig COLVI contributes to *Sgg* binding to de-matrix. HT29 and HCT116 cells were transfected with control or COL6A1 siRNA. Dc-matrices were prepared as described in the Materials and Methods section. Bacterial binding to the dc-matrices was then determined as described for binding to purified collagens. Bound bacteria were detected using crystal violet, as described in the Materials and Methods section. Mean + SEM is presented. Paired t test was used for statistical comparison. ****, p < 0.0001.
(TIF)

S11 Fig Dc-matrices derived from different *Sgg*-treated cells have varied effect in supporting cell proliferation. This was carried out as described in the Materials and Methods section and in the legend for Fig 4B and 4C. Briefly, HT29 (A) and HCT116 (B) cells were transiently transfected with control siRNA or COL6A1 siRNA and then co-cultured with the indicated Sgg strains or media only. Bacteria and cells were then eliminated to prepare dc-matrix. Fresh HT29 and HCT116 cells were seeded on the indicated dc-matrices and incubated for 24 hours. Cell proliferation was examined using the CCK-8 assay. Each experiment was repeated three times. Data is presented as the mean + SEM. Statistical analysis was done using unpaired, two-tailed t test. ****, p < 0.0001.
(TIF)
