# Supplementary material for: Modulation of the extracellular matrix by Streptococcus gallolyticus subsp. gallolyticus and importance in cell proliferation
Source: PLoS Pathog. 2022 Oct 3;18(10):e1010894. doi: 10.1371/journal.ppat.1010894 (PMC9560553; doi:10.1371/journal.ppat.1010894)
Supplement: S2 Table — a HT29 cells were cultured in the presence or absence of Sgg strain TX20005 for 24 hours (3 biological replicates). The cells were washed, lysed and digested in ammonium bicarbonate buffer and trypsin. The resulting peptide mixtures were analyzed by a nanoLC-1200 system coupled to an Orbitrap Fusion Lumos mass spectrometer. Only the collagen types that show increase in all three biological replicates are listed. b Relative protein abundance is shown as the mean iFOT ± SEM. iFOT is the normalization of individual protein intensity to the total protein intensity within one experiment. c Protein not detected. The limit of detection is equivalent to iFOT = 0.0005. d Only detected in one biological replicate. e Undetermined. Statistical comparison between HT29 + Sgg and HT29 was not performed for these samples due to the fact that the corresponding collagen peptide chains in untreated HT29 were not detected. (DOCX) [file ppat.1010894.s002.docx]

**Supplemental Table S2. Relative abundance of several types of collagens in whole cell lysates analyzed by Mass Spectrometry** ^a^**.**

|  | **HT29**^b^ | **HT29 + *Sgg***^b^ | **P value** |
| --- | --- | --- | --- |
| COL6A3 | ND^c^ | 11.5 ± 3.1^e^ | UD^e^ |
| COL6A1 | ND^c^ | 4.6 ± 1.3 | UD^e^ |
| COL3A1 | ND^c^ | 3.9 ± 1.5 | UD^e^ |
| COL4A2 | ND^c^ | 2.9 ± 0.9 | UD^e^ |
| COL1A1 | 0.02^d^ | 0.8 ± 0.5 | 0.057 |
