# Supplementary figures and images for: Modulation of the extracellular matrix by Streptococcus gallolyticus subsp. gallolyticus and importance in cell proliferation
Source: PLoS Pathog. 2022 Oct 3;18(10):e1010894. doi: 10.1371/journal.ppat.1010894 (PMC9560553; doi:10.1371/journal.ppat.1010894)

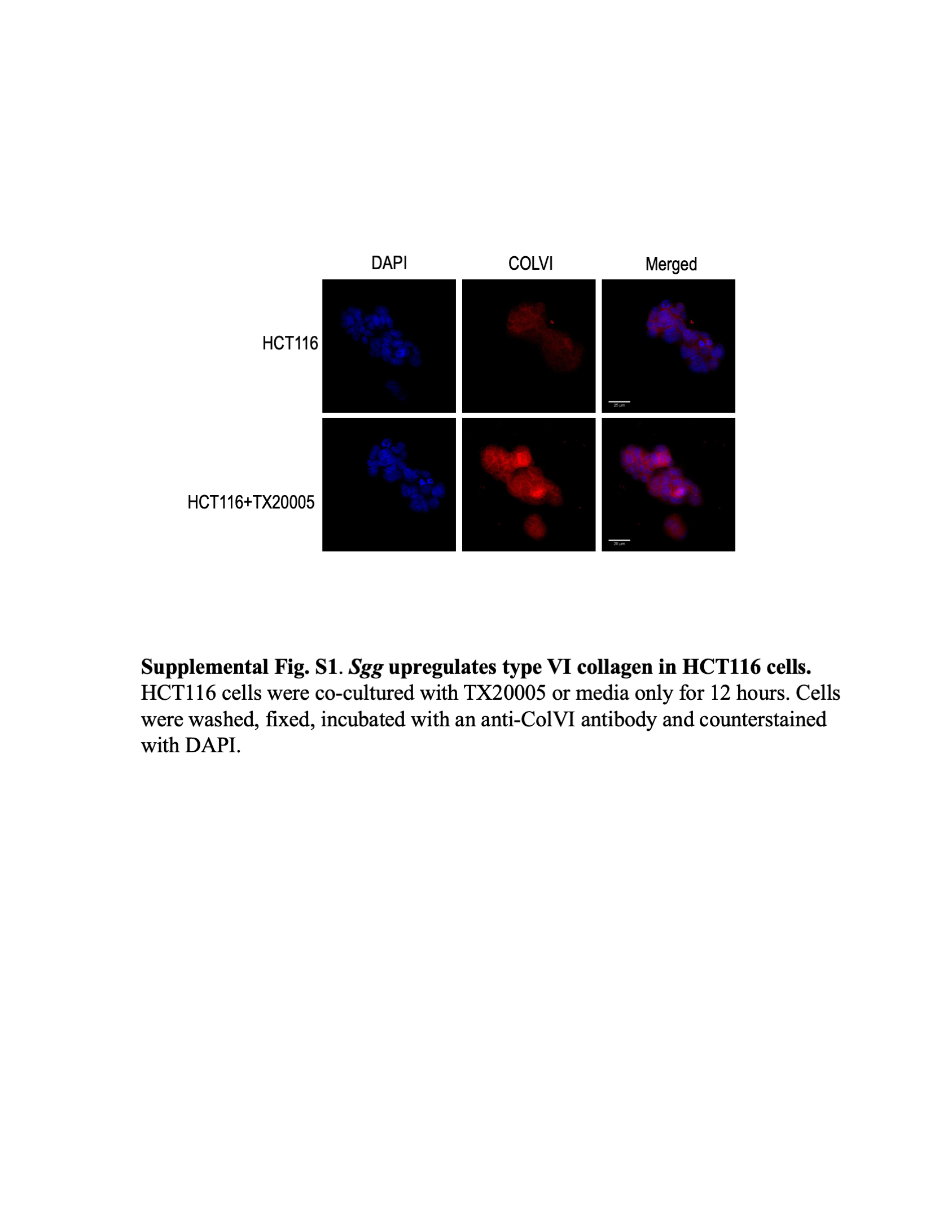

Supplement: S1 Fig — HCT116 cells were co-cultured with TX20005 or media only for 12 hours. Cells were washed, fixed, incubated with an anti-ColVI antibodv and counterstained with DAPI. (TIF) [file ppat.1010894.s003.tif]

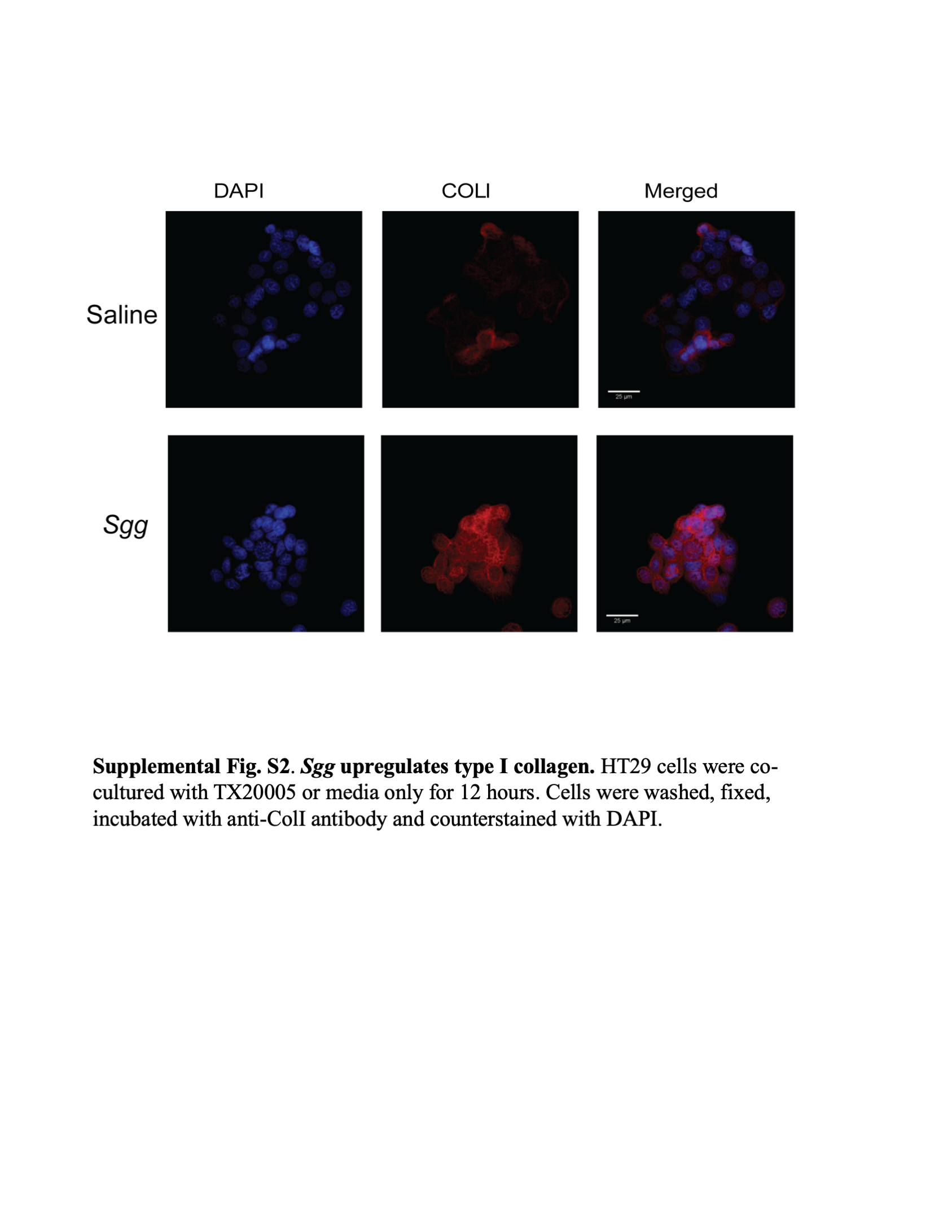

Supplement: S2 Fig — HT29 cells were co-cultured with TX20005 or media only for 12 hours. Cells were washed. fixed. incubated with anti-Coll antibody and counterstained with DAPI. (TIF) [file ppat.1010894.s004.tif]

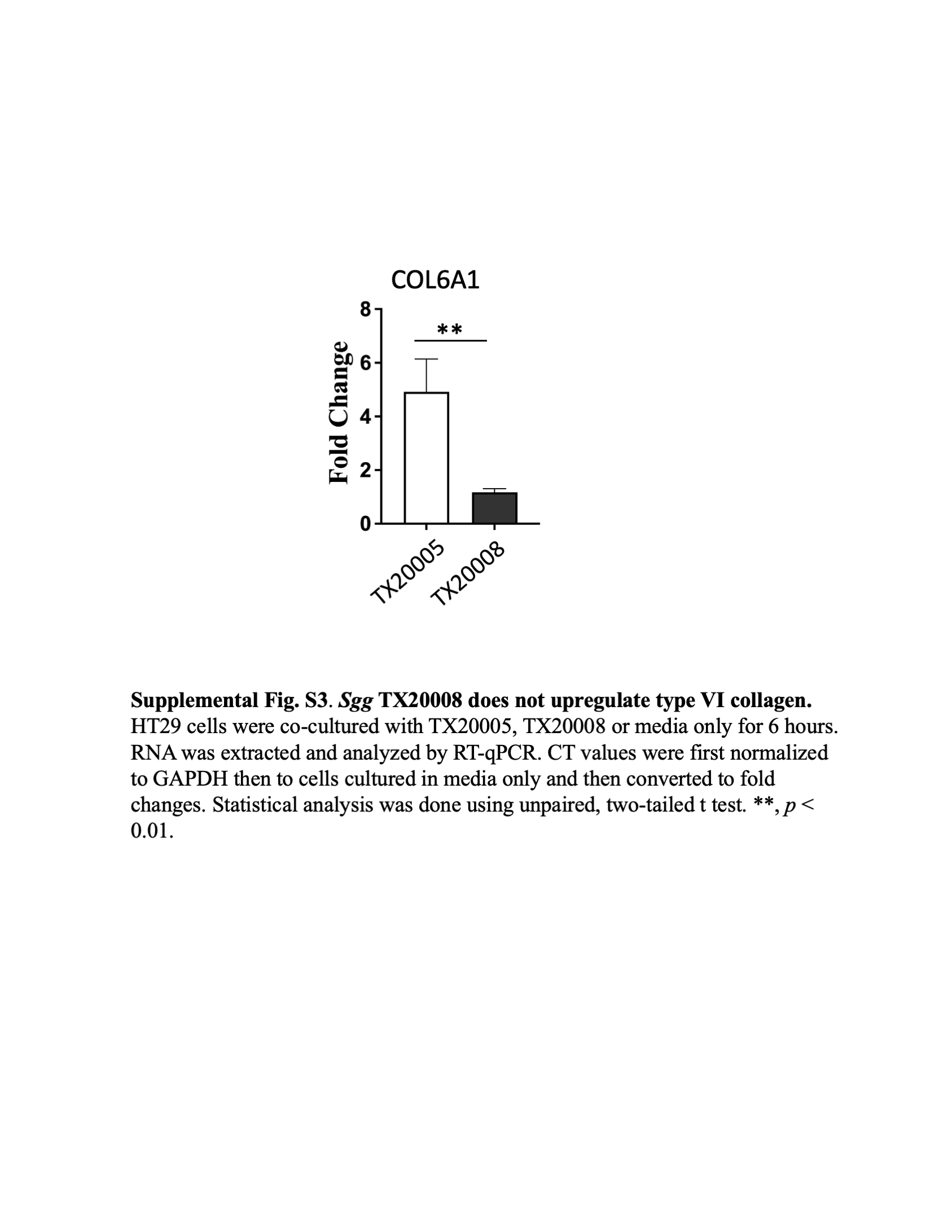

Supplement: S3 Fig — HT29 cells were co-cultured with TX20005, TX20008 or media only for 6 hours. RNA was extracted and analyzed by RT-qPCR. CT values were first normalized to GAPDH then to cells cultured in media only and then converted to fold changes. Statistical analysis was done using unpaired, two-tailedt test. **, p<0.01. (TIF) [file ppat.1010894.s005.tif]

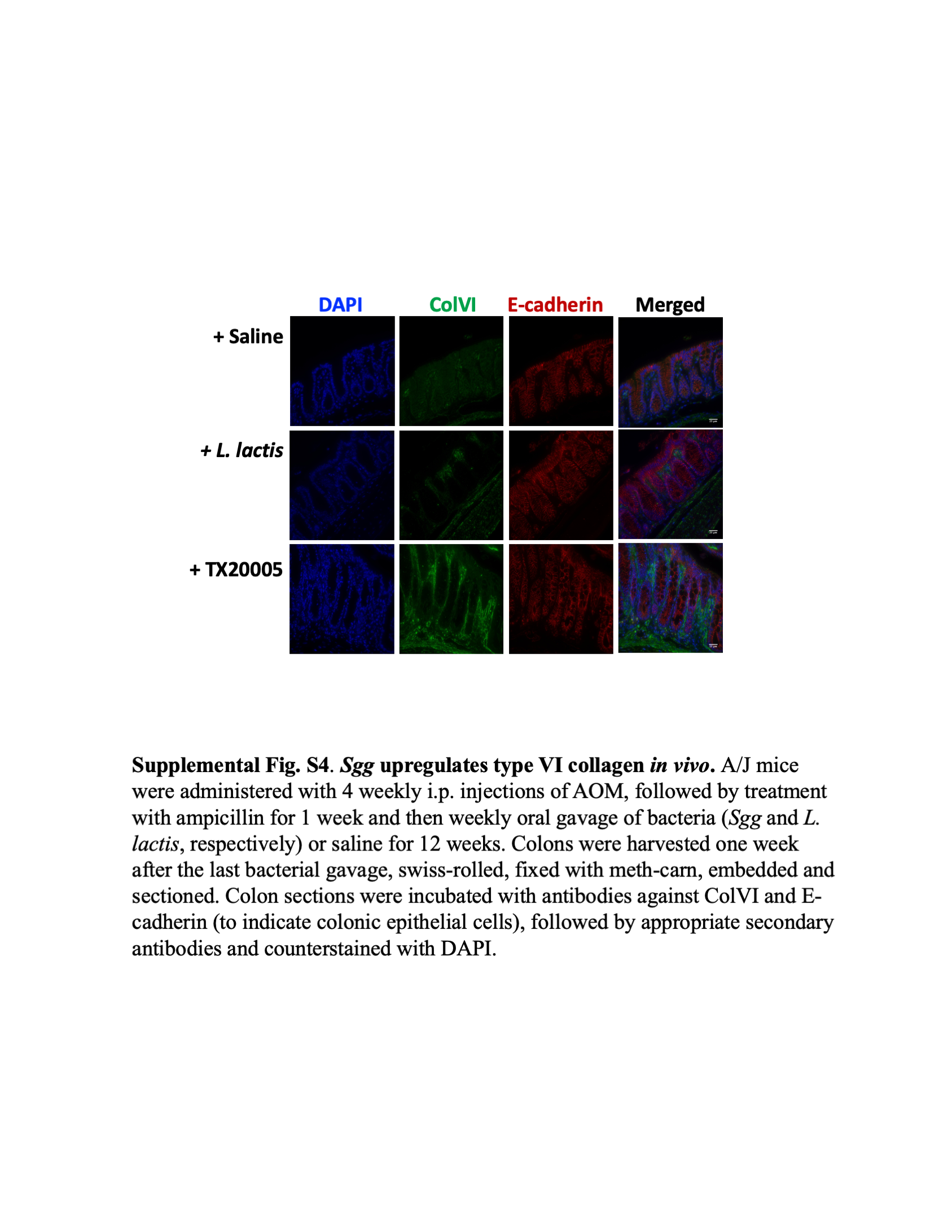

Supplement: S4 Fig — A/J mice were administered with 4 weekly i.p. injections of AOM, followed by treatment with ampicillin for 1 week and then weekly oral gavage of bacteria (Sgg and L. lactis, respectively) or saline for 12 weeks. Colons were harvested one week after the last bacterial gavage, swiss-rolled, fixed with meth-carn, embedded and sectioned. Colon sections were incubated with antibodies against ColVI and E- cadherin (to indicate colonic epithelial cells), followed by appropriate secondary antibodies and counterstained with DAPI. (TIF) [file ppat.1010894.s006.tif]

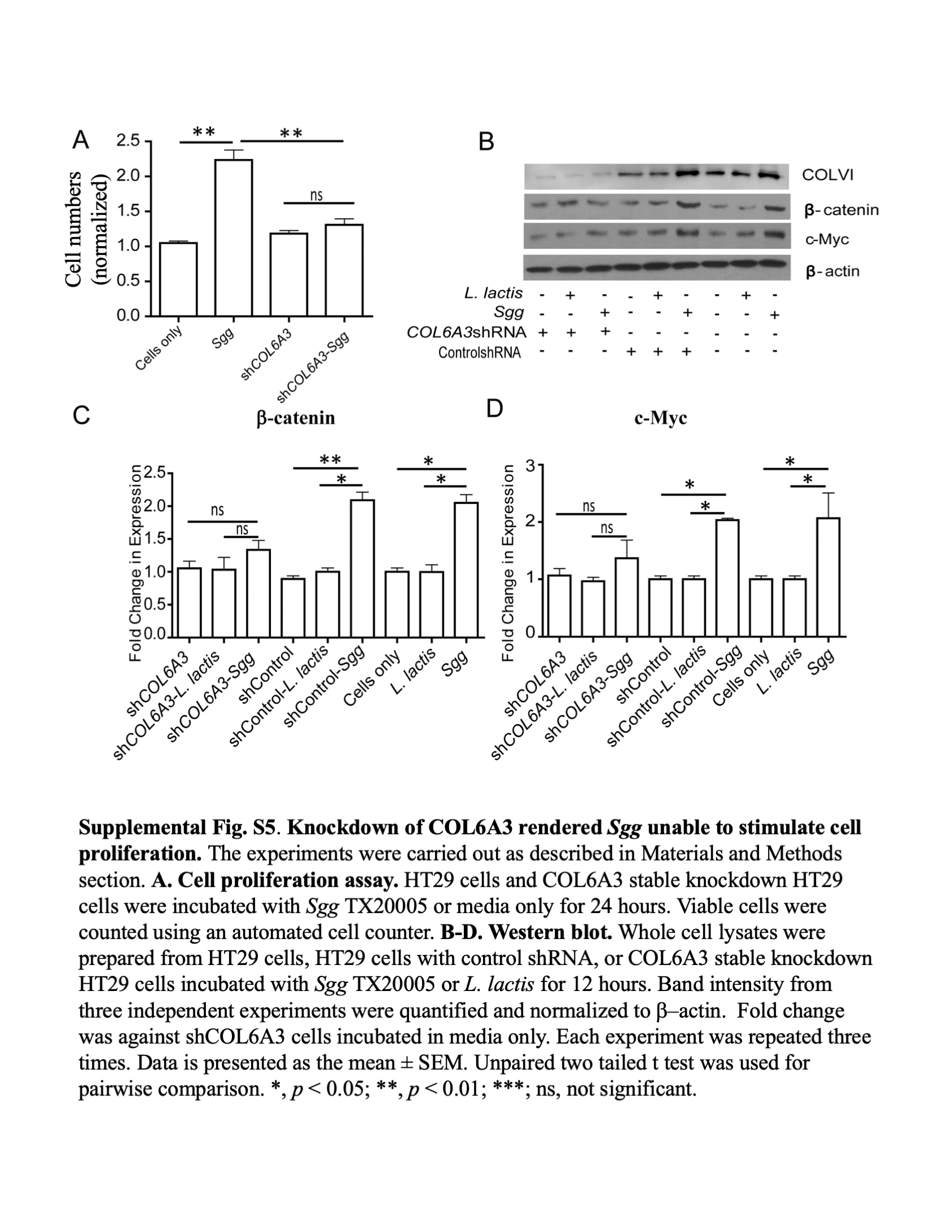

Supplement: S5 Fig — The experiments were carried out as described in Materials and Methods section. A. Cell proliferation assay. HT29 cells and COL6A3 stable knockdown HT29 cells were incubated with gg TX20005 or media only for 24 hours. Viable cells were counted using an automated cell counter. B-D. Western blot. Whole cell lysates were prepared from HT29 cells, HT29 cells with control shRNA, or COL6A3 stable knockdown HT29 cells incubated with Sgg TX20005 or L. lactis for 12 hours. Band intensity from three independent experiments were quantified and normalized to -actin. Fold change was against shCOL6A3 cells incubated in media only. Each experiment was repeated three times. Data is presented as the mean + SEM. Unpaired two tailed t test was used for pairwise comparison. *, p <0.05; **, p <0.01; ***; ns, not significant. (TIF) [file ppat.1010894.s007.tif]

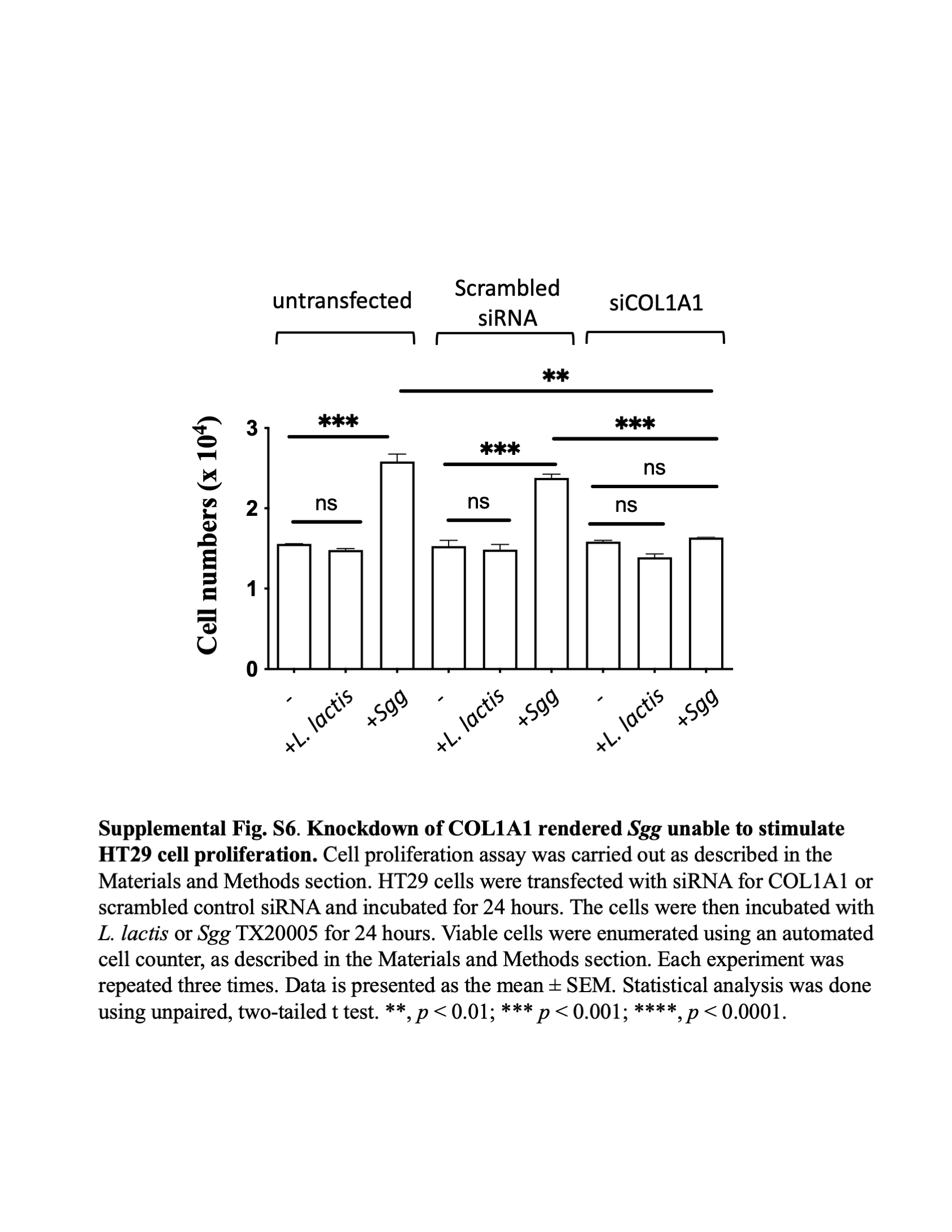

Supplement: S6 Fig — Cell proliferation assay was carried out as described in the Materials and Methods section. HT29 cells were transfected with siRNA for COL1A1 or scrambled control siRNA and incubated for 24 hours. The cells were then incubated with L. lactis or gg TX20005 for 24 hours. Viable cells were enumerated using an automated cell counter, as described in the Materials and Methods section. Each experiment was repeated three times. Data is presented as the mean + SEM. Statistical analysis was done using unpaired, two-tailed t test. **, p < 0.01; *** p <0.001; ****, p <0.0001. (TIF) [file ppat.1010894.s008.tif]

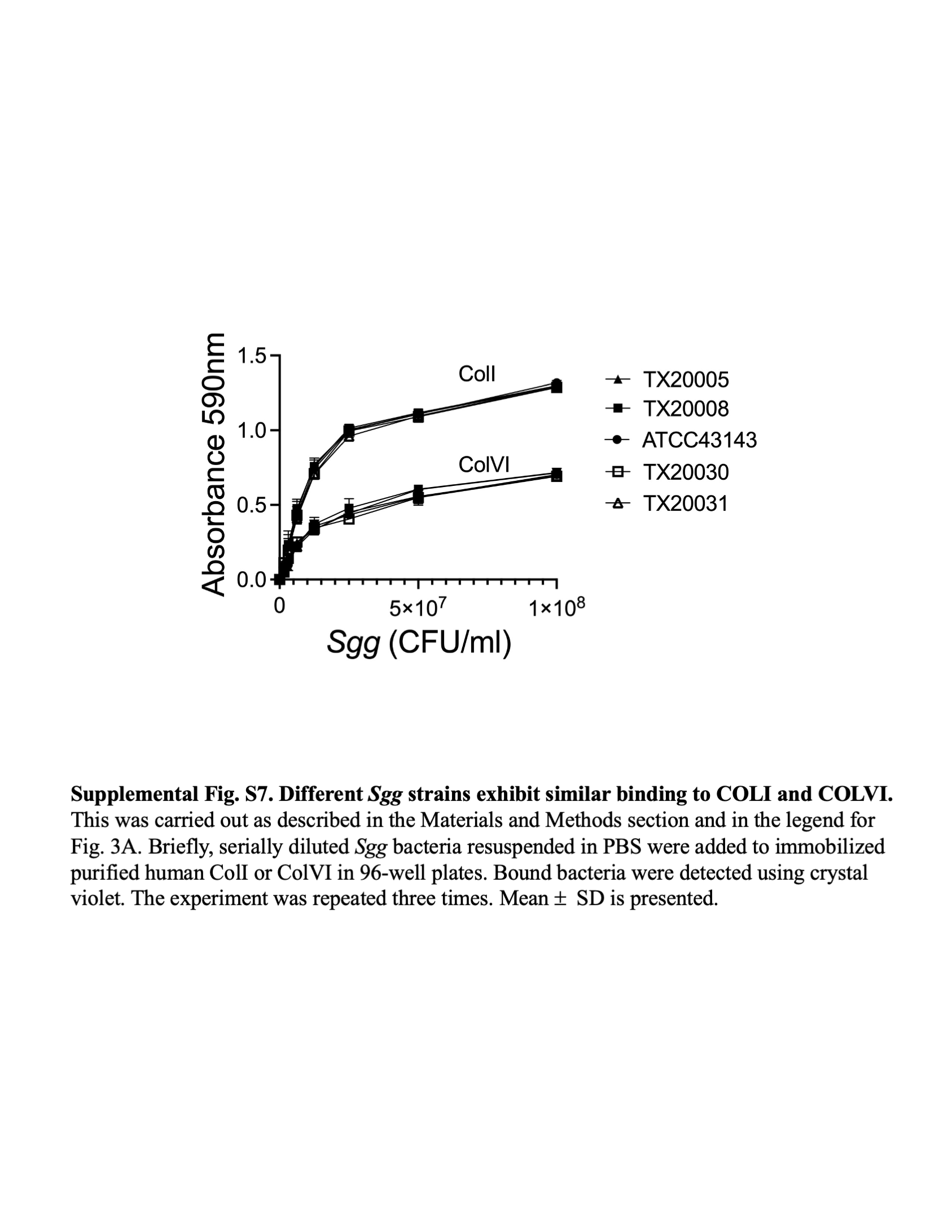

Supplement: S7 Fig — This was carried out as described in the Materials and Methods section and in the legend for Fig 3A. Briefly, serially diluted gg bacteria resuspended in PBS were added to immobilized purified human Coll or ColVI in 96-well plates. Bound bacteria were detected using crystal violet. The experiment was repeated three times. Mean + SD is presented. (TIF) [file ppat.1010894.s009.tif]

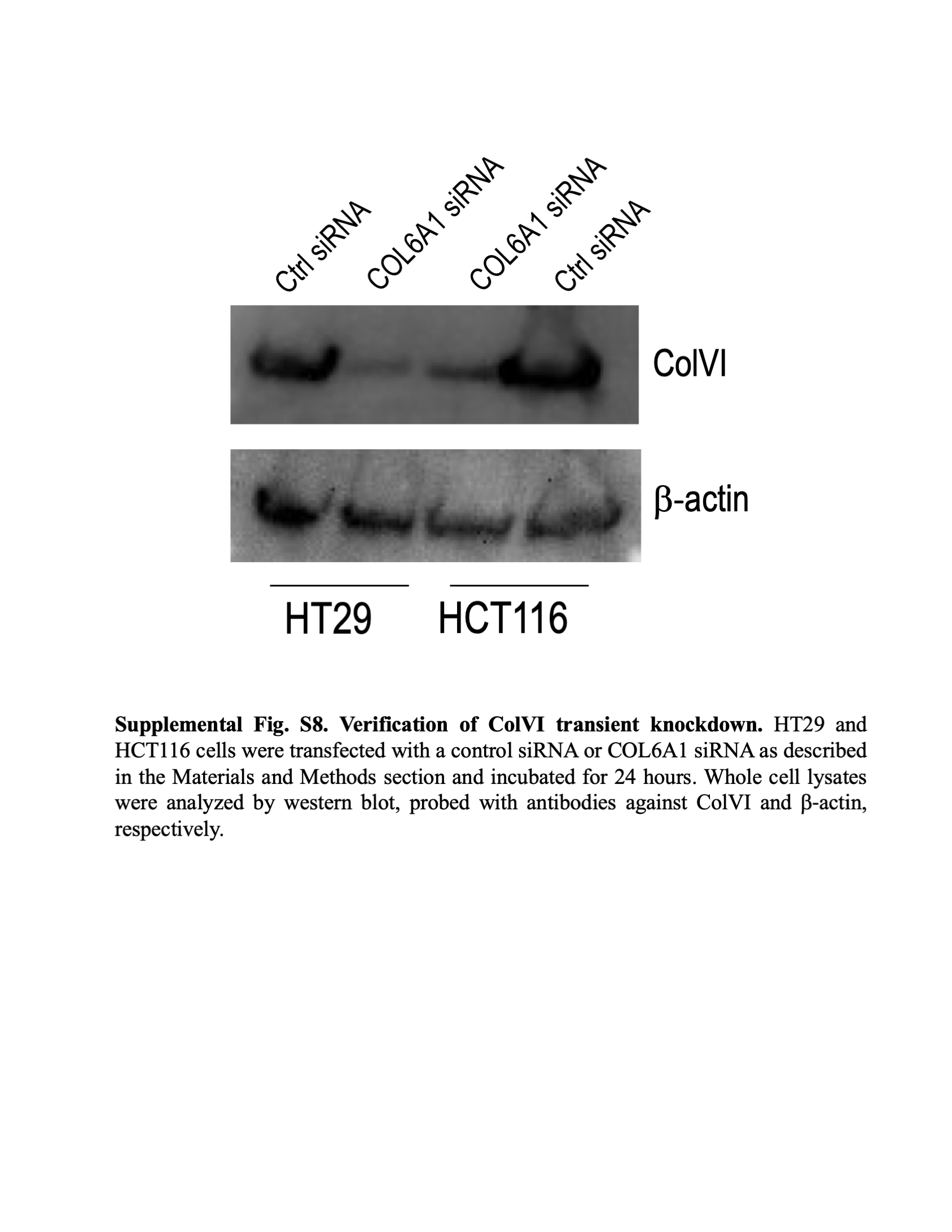

Supplement: S8 Fig — HT29 and HCT116 cells were transfected with a control siRNA or COL6A1 siRNA as described in the Materials and Methods section and incubated for 24 hours. Whole cell lysates were analyzed by western blot, probed with antibodies against ColVI and ß-actin, respectively. (TIF) [file ppat.1010894.s010.tif]

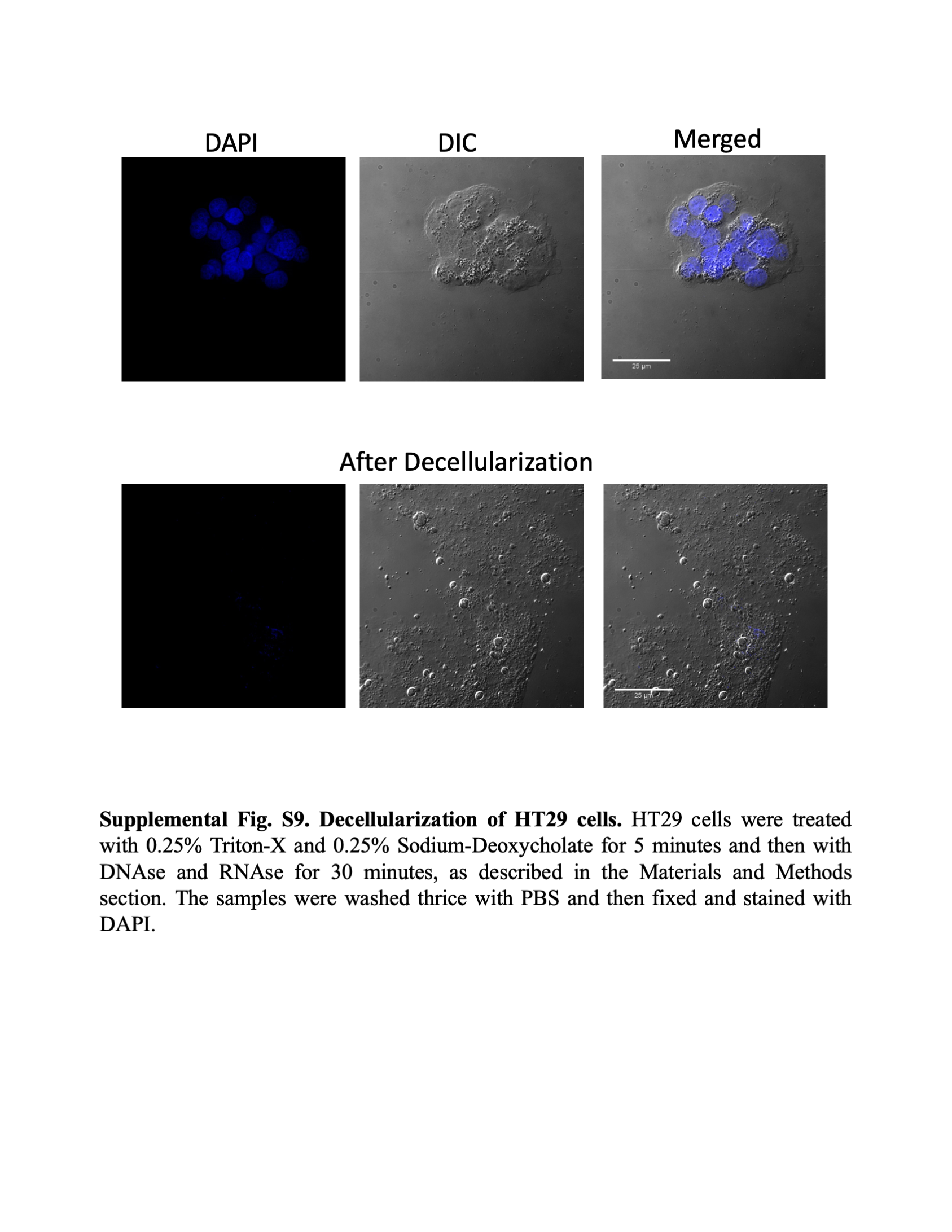

Supplement: S9 Fig — HT29 cells were treated with 0.25% Triton-X and 0.25% Sodium-Deoxycholate for 5 minutes and then with DNAse and RNAse for 30 minutes, as described in the Materials and Methods section. The samples were washed thrice with PBS and then fixed and stained with DAPI. (TIF) [file ppat.1010894.s011.tif]

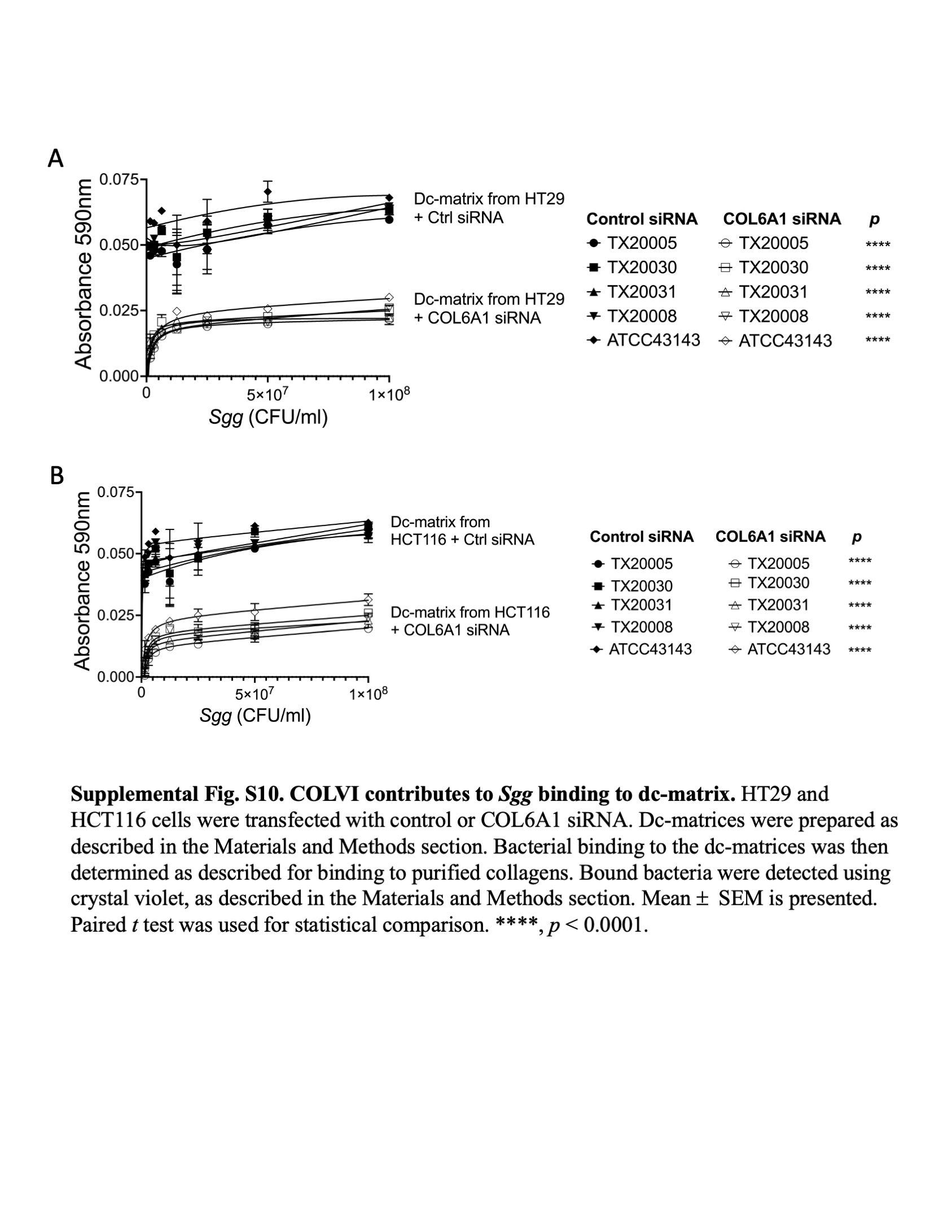

Supplement: S10 Fig — HT29 and HCT116 cells were transfected with control or COL6A1 siRNA. Dc-matrices were prepared as described in the Materials and Methods section. Bacterial binding to the dc-matrices was then determined as described for binding to purified collagens. Bound bacteria were detected using crystal violet, as described in the Materials and Methods section. Mean + SEM is presented. Paired t test was used for statistical comparison. ****, p < 0.0001. (TIF) [file ppat.1010894.s012.tif]

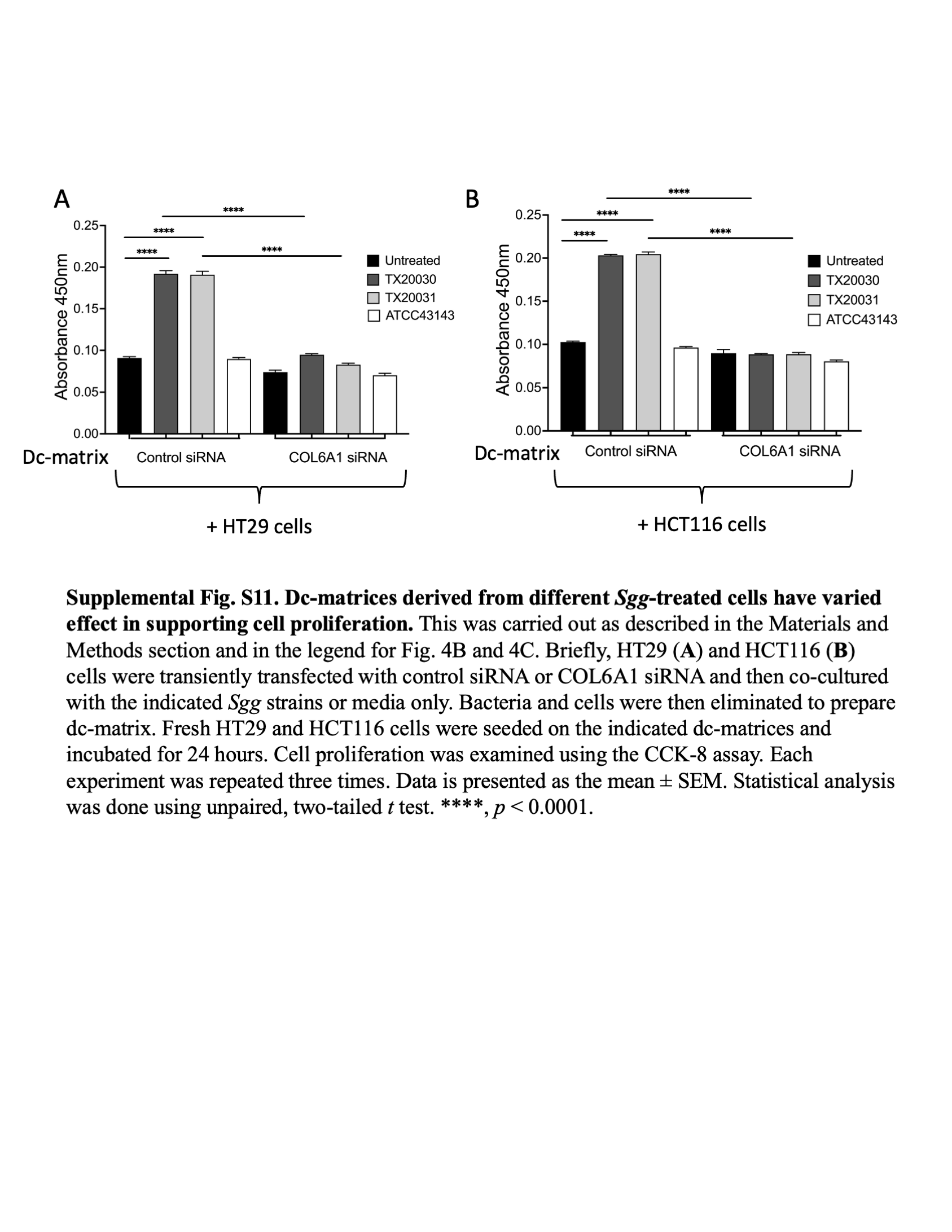

Supplement: S11 Fig — This was carried out as described in the Materials and Methods section and in the legend for Fig 4B and 4C. Briefly, HT29 (A) and HCT116 (B) cells were transiently transfected with control siRNA or COL6A1 siRNA and then co-cultured with the indicated Sgg strains or media only. Bacteria and cells were then eliminated to prepare dc-matrix. Fresh HT29 and HCT116 cells were seeded on the indicated dc-matrices and incubated for 24 hours. Cell proliferation was examined using the CCK-8 assay. Each experiment was repeated three times. Data is presented as the mean + SEM. Statistical analysis was done using unpaired, two-tailed t test. ****, p < 0.0001. (TIF) [file ppat.1010894.s013.tif]
